# Author Correction: Climatic and tectonic drivers shaped the tropical distribution of coral reefs

**DOI:** 10.1038/s41467-022-31650-4

**Published:** 2022-07-07

**Authors:** Lewis A. Jones, Philip D. Mannion, Alexander Farnsworth, Fran Bragg, Daniel J. Lunt

**Affiliations:** 1 Centro de Investigación Mariña, Grupo de Ecoloxía Animal, Universidade de Vigo, 36310 Vigo, Spain; 2 Department of Earth Sciences, University College London, Gower Street, London, WC1E 6BT UK; 3 School of Geographical Sciences, University of Bristol, Bristol, BS8 1SS UK

**Keywords:** Macroecology, Palaeontology

Correction to: *Nature Communications* 10.1038/s41467-022-30793-8, published online 14 June 2022.

The original version of this Article contained an error in Figs. 4, 5, in which the x-axes labels read 10^6^ km^2^ instead of 10^5^ km^2^. This has been corrected in both the PDF and HTML versions of the Article.

The original version of the Supplementary Information associated with this Article contained an error in Supplementary Figures 6, 8, 11, in which the x-axes labels read 10^6^ km^2^ instead of 10^5^ km^2^. The HTML has been updated to include a corrected version of the Supplementary Information.

