# Supplementary material for: Climatic and tectonic drivers shaped the tropical distribution of coral reefs
Source: Nat Commun. 2022 Jun 14;13:3120. doi: 10.1038/s41467-022-30793-8 (PMC9198051; doi:10.1038/s41467-022-30793-8)
Supplement: Supplementary file 1 — Supplementary information [file 41467_2022_30793_MOESM1_ESM.pdf]

Supplementary Material for

Climatic and tectonic drivers shaped the tropical distribution of coral reefs

Lewis A. Jones[1], **Philip D. Mannion**[2], **Alexander Farnsworth**[3], **Fran Bragg**[3] **and Daniel J. Lunt**[3]

[1]Centro de Investigación Mariña, Grupo de Ecoloxía Animal, Universidade de Vigo, 36310, Spain
[2]Department of Earth Sciences, University College London, Gower Street, WC1E 6BT, UK
[3]School of Geographical Sciences, University of Bristol, Bristol, BS8 1TH, UK
Author for correspondence: Lewis A. Jones (LewisAlan.Jones@uvigo.es)

This PDF includes:

- Supplementary Tables
- Supplementary Figures

Supplementary Tables

Table S1. Full list of the palaeoclimate simulations, and respective CO_2 concentration, used in this study. Climate simulations were performed using the HadCM3BL-M2.1aE (see Methods for details). Stage ages are given in million years before present (Ma). Simulation codes refer to the specific simulation used and the unique identifier it is stored under on the BRIDGE system (http://www.bridge.bris.ac.uk/resources/simulations).

Simulation	Stage	Max Ma	Mid Ma	Min Ma	CO_2 (ppmv)
tdwge	Pre-industrial	0.00	0.00	0.00	280
tdqpq	Piacenzian	3.60	3.09	2.59	400
tdqpp	Zanclean	5.33	4.47	3.60	400
tdqpo	Messinian	7.25	6.29	5.33	400
tdqpn	Tortonian	11.62	9.43	7.25	400
tdqpm	Serravallian	13.82	12.72	11.62	400
tdqpl	Langhian	15.97	14.90	13.82	400
tdqpk	Burdigalian	20.44	18.21	15.97	400
tdqpj	Aquitanian	23.03	21.74	20.44	400
tdluq	Chattian	28.10	25.57	23.03	560
tdlup	Rupelian	33.90	31.00	28.10	560
tdluk	Priabonian	38.00	35.95	33.90	1120
tdluj	Bartonian	41.30	39.65	38.00	1120
tdlur	Lutetian	47.80	44.55	41.30	1120
tdlud	Ypresian	56.00	51.90	47.80	1120
tdluc	Thanetian	59.20	57.60	56.00	1120
tdlub	Selandian	61.60	60.40	59.20	1120
tdlua	Danian	66.00	63.80	61.60	1120
tdihb	Maastrichtian	72.10	69.05	66.00	1120
tdpwc	Campanian	83.60	77.85	72.10	1120
tdpwd	Santonian	86.30	84.95	83.60	1120
tdpwe	Coniacian	89.80	88.05	86.30	1120
tdpwf	Turonian	93.90	91.85	89.80	1120
tdpwg	Cenomanian	100.50	97.20	93.90	1120
tdpwh	Albian	113.00	106.75	100.50	1120
tdpwi	Aptian	125.00	119.00	113.00	1120
tdpwj	Barremian	129.40	127.20	125.00	1120
tdpwk	Hauterivian	132.90	131.15	129.40	1120
tdpwl	Valanginian	139.80	136.35	132.90	1120
tdihm	Berriasian	145.00	142.40	139.80	1120
teqvq	Tithonian	152.10	148.55	145.00	1120
tdzoj	Kimmeridgian	157.30	154.70	152.10	1120
teqvr	Oxfordian	163.50	160.40	157.30	1120
teqvs	Callovian	166.10	164.80	163.50	1120
teqvt	Bathonian	168.30	167.20	166.10	1120
teqvu	Bajocian	170.30	169.30	168.30	1120
teqvz	Aalenian	174.10	172.20	170.30	1120
teqvw	Toarcian	182.70	178.40	174.10	1120
tdzoc	Pliensbachian	190.80	186.75	182.70	1120
tdzob	Sinemurian	199.30	195.05	190.80	1120
tdzoa	Hettangian	201.30	200.30	199.30	1120
teqva	Rhaetian	208.50	204.90	201.30	1120
teqvb	Norian	228.00	218.25	208.50	1120
teqvc	Carnian	237.00	232.50	228.00	1120
teqvd	Ladinian	242.00	239.50	237.00	1120
teqve	Anisian	247.20	244.60	242.00	1120

Table S2. The predictive performance of stage-level model projections for binary threshold: least training presence. Results show the percentage of fossil coral reef localities intersecting with cells predicted to be suitable (standard/buffered), and those randomly generated. Buffered results include a one-cell search radius in all directions of the reef locality to account for uncertainty in palaeorotations. Reported values for the random predictive performance is the mean percentage of predictive accuracy from 1000 sets of *n* points, where *n* is the number of observed reef localities for each stage. One-sample Wilcoxon signed-rank test results (V statistic; *P*) imply that the majority of model hindcasts performed better than expected at random. Non-significant results are denoted in bold ($P > 0.05$).

				**Predictive performance (%)**				
**Stage**	**True positives**	**False negatives**	**Localities**	**Standard**	**Buffered**	**Random**	**V**	***P***
Piacenzian	10	0	10	100.00	100.00	4.39	0	< 0.001
Zanclean	16	1	17	94.12	94.12	4.88	0	< 0.001
Messinian	12	1	13	92.31	92.31	4.63	0	< 0.001
Tortonian	25	11	36	69.44	80.56	4.66	0	< 0.001
Serravallian	5	2	7	71.43	71.43	5.27	0	< 0.001
Langhian	7	3	10	70.00	70.00	4.79	0	< 0.001
Burdigalian	20	7	27	74.07	74.07	5.17	0	< 0.001
Aquitanian	11	3	14	78.57	78.57	5.01	0	< 0.001
Chattian	11	1	12	91.67	91.67	5.36	0	< 0.001
Rupelian	6	2	8	75.00	75.00	4.70	0	< 0.001
Priabonian	11	2	13	84.62	100.00	5.42	0	< 0.001
Bartonian	0	1	1	0.00	0.00	7.90	3160	**1**
Lutetian	5	1	6	83.33	83.33	6.65	0	< 0.001
Ypresian	4	0	4	100.00	100.00	6.08	0	< 0.001
Thanetian	8	5	13	61.54	84.62	6.41	0	< 0.001
Selandian	2	0	2	100.00	100.00	6.50	0	< 0.001
Danian	8	2	10	80.00	80.00	6.00	0	< 0.001
Maastrichtian	8	0	8	100.00	100.00	6.79	0	< 0.001
Campanian	9	1	10	90.00	90.00	6.58	0	< 0.001
Santonian	2	0	2	100.00	100.00	6.80	0	< 0.001
Coniacian	2	0	2	100.00	100.00	6.00	0	< 0.001
Turonian	4	3	7	57.14	71.43	6.87	0	< 0.001
Cenomanian	7	0	7	100.00	100.00	6.60	0	< 0.001
Albian	15	0	15	100.00	100.00	5.88	0	< 0.001
Aptian	18	0	18	100.00	100.00	5.67	0	< 0.001
Barremian	5	2	7	71.43	100.00	5.06	0	< 0.001
Hauterivian	7	0	7	100.00	100.00	4.91	0	< 0.001
Valanginian	6	0	6	100.00	100.00	4.27	0	< 0.001
Berriasian	6	0	6	100.00	100.00	5.07	0	< 0.001
Tithonian	24	3	27	88.89	88.89	6.28	0	< 0.001
Kimmeridgian	39	1	40	97.50	97.50	6.16	0	< 0.001
Oxfordian	36	10	46	78.26	84.78	6.40	0	< 0.001
Callovian	4	0	4	100.00	100.00	6.08	0	< 0.001
Bathonian	4	0	4	100.00	100.00	5.90	0	< 0.001
Bajocian	21	0	21	100.00	100.00	5.14	0	< 0.001
Aalenian	4	0	4	100.00	100.00	4.75	0	< 0.001
Toarcian	1	0	1	100.00	100.00	5.80	0	< 0.001
Pliensbachian	5	0	5	100.00	100.00	3.90	0	< 0.001
Sinemurian	4	2	6	66.67	66.67	4.75	0	< 0.001
Hettangian	2	0	2	100.00	100.00	4.70	0	< 0.001
Rhaetian	12	4	16	75.00	75.00	4.58	0	< 0.001
Norian	33	4	37	89.19	89.19	4.39	0	< 0.001
Carnian	12	1	13	92.31	92.31	5.08	0	< 0.001
Ladinian	8	0	8	100.00	100.00	4.68	0	< 0.001
Anisian	3	0	3	100.00	100.00	4.57	0	< 0.001

Table S3. The predictive performance of stage-level model projections for binary threshold: maximising the sum of sensitivity and specificity. Results show the percentage of fossil coral reef localities intersecting with cells predicted to be suitable (standard/buffered), and those randomly generated. Buffered results include a one-cell search radius in all directions of the reef locality to account for uncertainty in palaeorotations. Reported values for the random predictive performance is the mean percentage of predictive accuracy from 1000 sets of *n* points, where *n* is the number of observed reef localities for each stage. One-sample Wilcoxon signed-rank test results (V statistic; *P*) imply that the majority of model hindcasts performed better than expected at random. Non-significant results are denoted in bold (*P* > 0.05).

	True	False		Predictive performance (%)				
**Stage**	**positives**	**negatives**	**Localities**	**Standard**	**Buffered**	**Random**	**V**	***P***
Piacenzian	8	2	10	80.00	80.00	3.58	0	< 0.001
Zanclean	15	2	17	88.24	88.24	3.94	0	< 0.001
Messinian	5	8	13	38.46	38.46	3.48	0	< 0.001
Tortonian	10	26	36	27.78	27.78	3.84	0	< 0.001
Serravallian	4	3	7	57.14	57.14	3.89	0	< 0.001
Langhian	4	6	10	40.00	40.00	3.61	0	< 0.001
Burdigalian	13	14	27	48.15	48.15	3.76	0	< 0.001
Aquitanian	5	9	14	35.71	35.71	3.67	0	< 0.001
Chattian	9	3	12	75.00	75.00	3.74	0	< 0.001
Rupelian	3	5	8	37.50	37.50	3.58	0	< 0.001
Priabonian	6	7	13	46.15	46.15	4.75	0	< 0.001
Bartonian	0	1	1	0.00	0.00	5.30	1431	**1**
Lutetian	4	2	6	66.67	66.67	4.88	0	< 0.001
Ypresian	2	2	4	50.00	50.00	5.75	0	< 0.001
Thanetian	5	8	13	38.46	38.46	4.88	0	< 0.001
Selandian	1	1	2	50.00	50.00	4.90	453	**1**
Danian	4	6	10	40.00	50.00	4.55	0	< 0.001
Maastrichtian	8	0	8	100.00	100.00	5.81	0	< 0.001
Campanian	6	4	10	60.00	60.00	4.80	0	< 0.001
Santonian	2	0	2	100.00	100.00	5.30	0	< 0.001
Coniacian	2	0	2	100.00	100.00	5.15	0	< 0.001
Turonian	3	4	7	42.86	42.86	5.19	0	< 0.001
Cenomanian	6	1	7	85.71	100.00	5.40	0	< 0.001
Albian	12	3	15	80.00	80.00	4.84	0	< 0.001
Aptian	14	4	18	77.78	83.33	4.22	0	< 0.001
Barremian	5	2	7	71.43	71.43	4.11	0	< 0.001
Hauterivian	5	2	7	71.43	71.43	4.07	0	< 0.001
Valanginian	4	2	6	66.67	66.67	3.82	0	< 0.001
Berriasian	6	0	6	100.00	100.00	3.63	0	< 0.001
Tithonian	18	9	27	66.67	74.07	4.46	0	< 0.001
Kimmeridgian	24	16	40	60.00	62.50	4.49	0	< 0.001
Oxfordian	21	25	46	45.65	52.17	4.71	0	< 0.001
Callovian	2	2	4	50.00	50.00	4.18	0	< 0.001
Bathonian	0	4	4	0.00	0.00	3.45	8646	**1**
Bajocian	7	14	21	33.33	42.86	3.67	0	< 0.001
Aalenian	2	2	4	50.00	50.00	3.40	0	< 0.001
Toarcian	1	0	1	100.00	100.00	3.60	0	< 0.001
Pliensbachian	5	0	5	100.00	100.00	3.04	0	< 0.001
Sinemurian	1	5	6	16.67	50.00	3.00	4626	**1**
Hettangian	0	2	2	0.00	0.00	2.40	1176	**1**
Rhaetian	7	9	16	43.75	43.75	3.43	0	< 0.001
Norian	26	11	37	70.27	72.97	3.35	0	< 0.001
Carnian	12	1	13	92.31	92.31	4.01	0	< 0.001
Ladinian	8	0	8	100.00	100.00	3.85	0	< 0.001
Anisian	3	0	3	100.00	100.00	3.90	0	< 0.001

Supplementary Figures

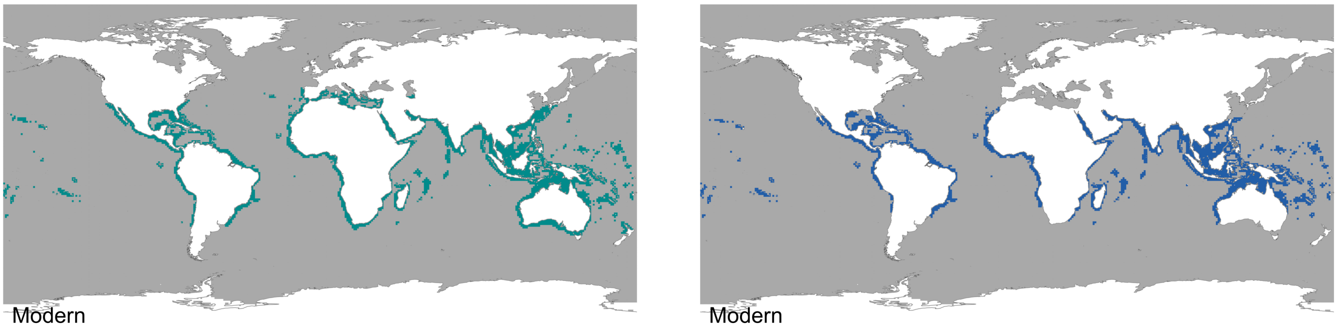

Figure S1. Binary habitat suitability maps (suitable/unsuitable) for modern coral reefs. Binary maps are converted from continuous suitability predictions using the ‘LTP’ (least training presence) and ‘MaxSSS’ (maximising the sum of sensitivity and specificity) thresholds. LTP predictions are on the left (suitable locations marked in green), while MaxSSS predictions are on the right (suitable locations marked in blue).

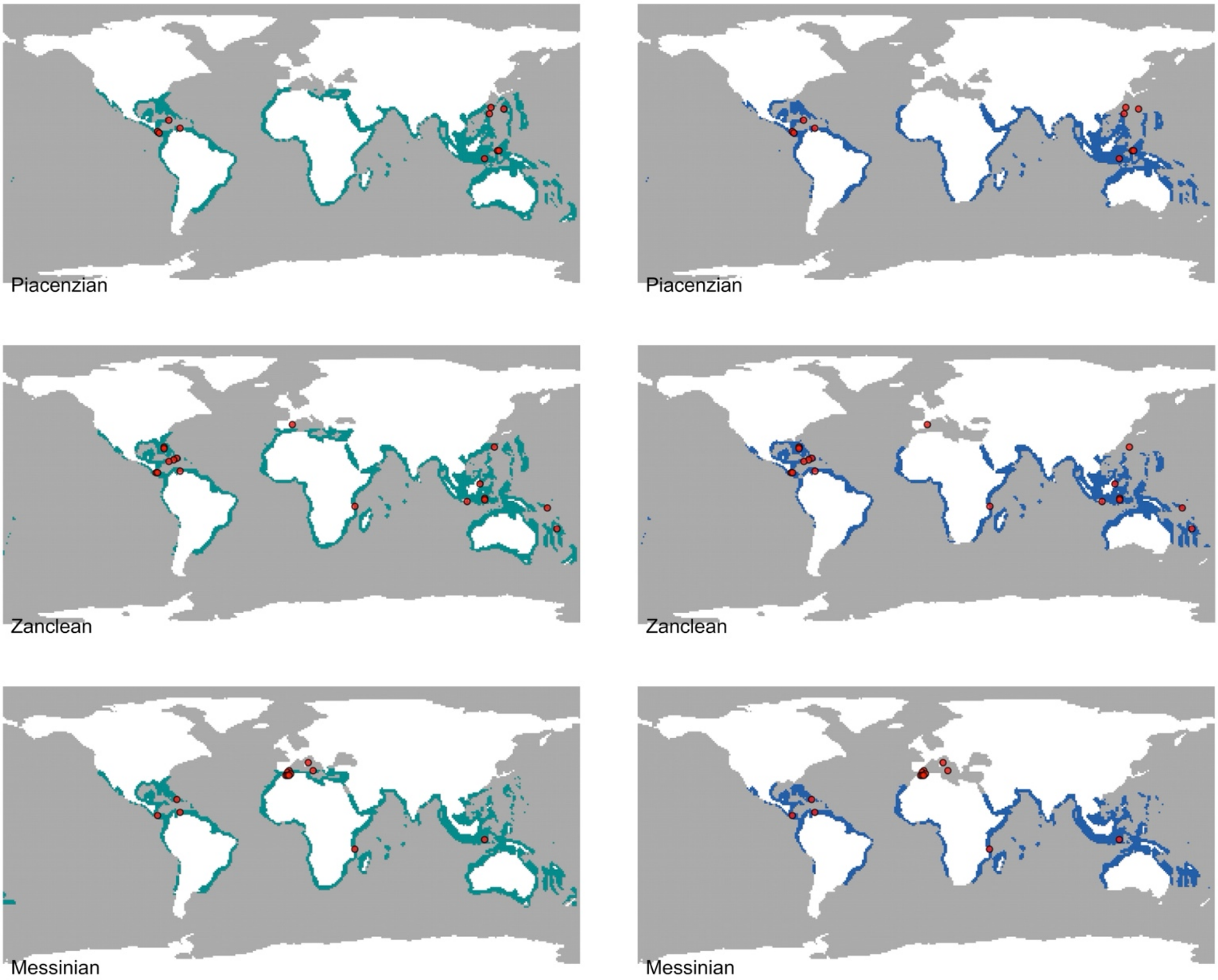

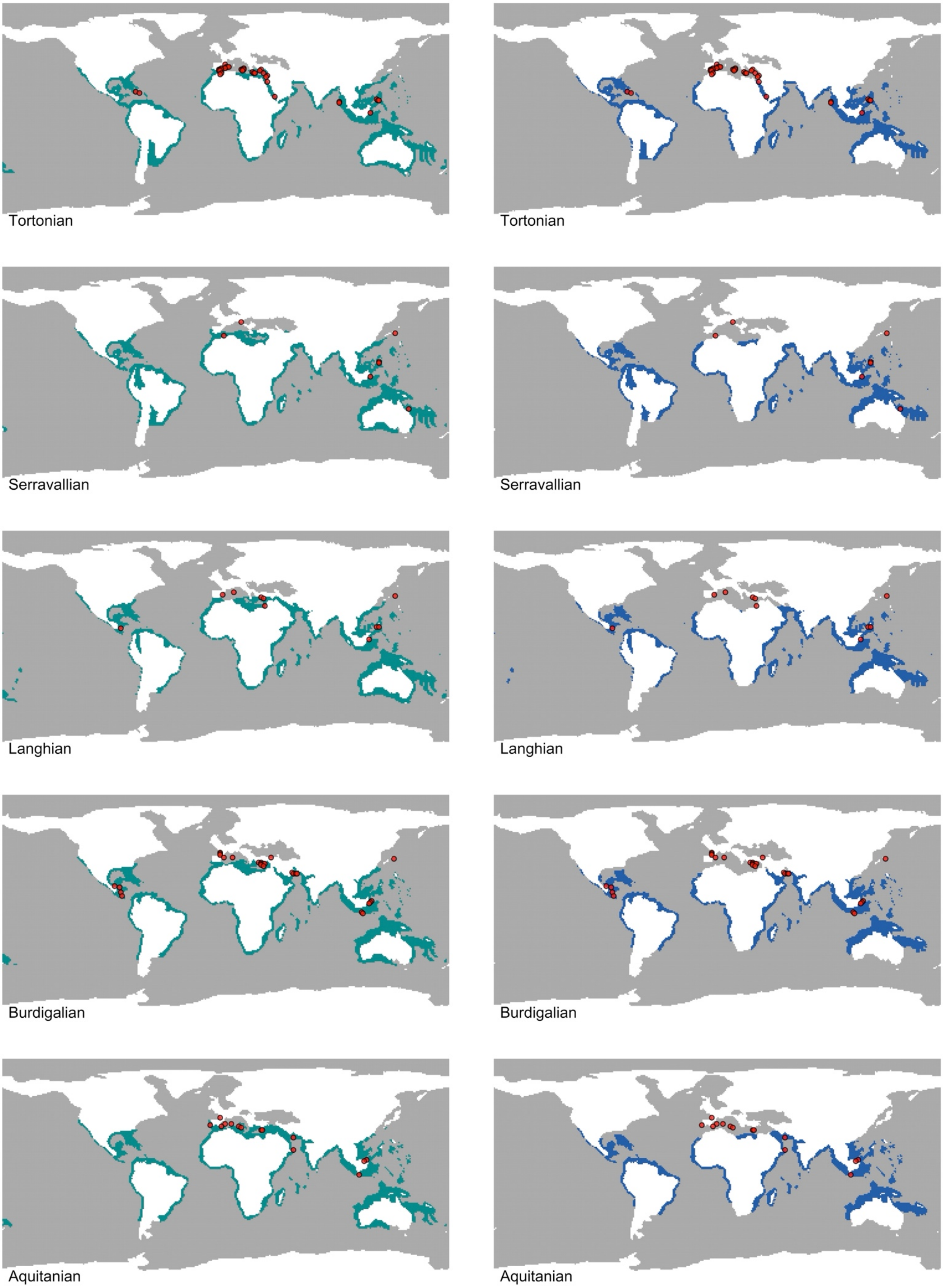
Tortonian
Tortonian
Serravallian
Serravallian
Langhian
Langhian
Burdigalian
Burdigalian
Aquitanian
Aquitanian

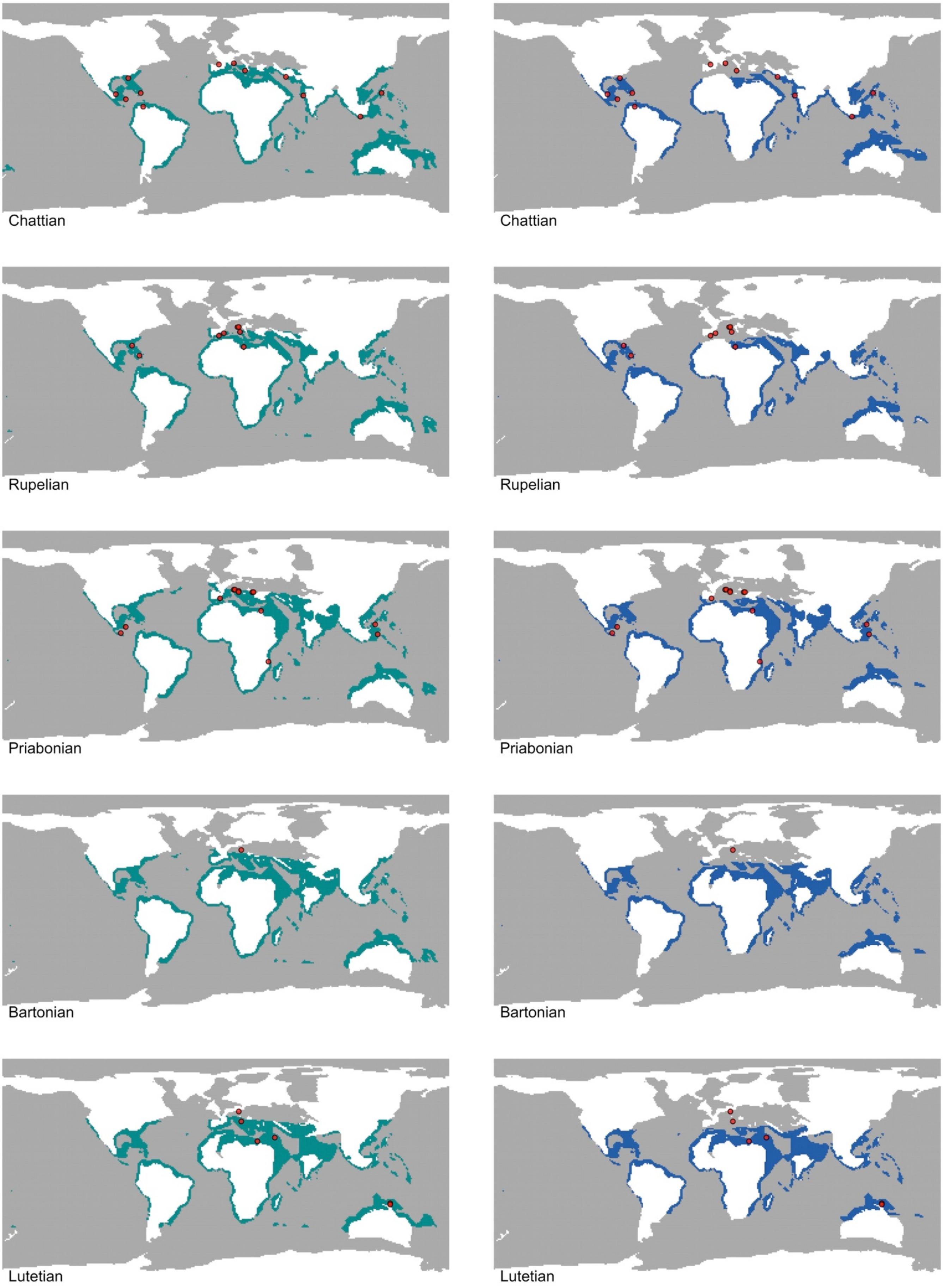
Chattian
Chattian
Rupelian
Rupelian
Priabonian
Priabonian
Bartonian
Bartonian
Lutetian
Lutetian

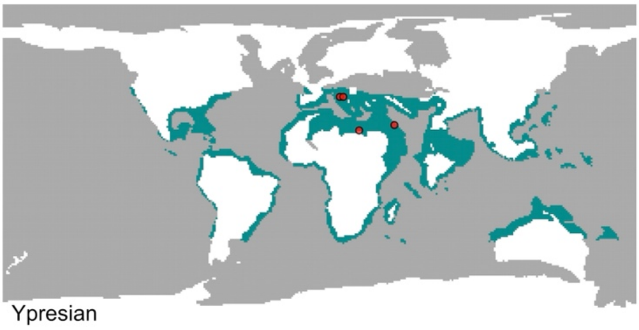

Ypresian

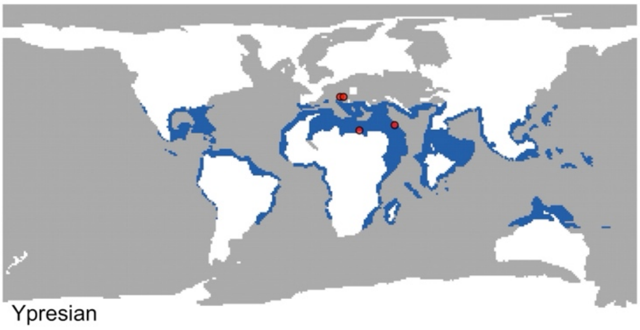

Ypresian

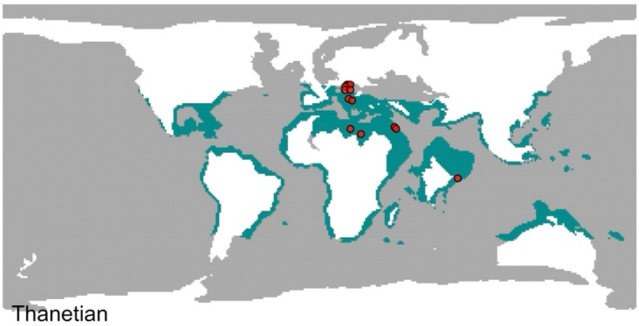

Thanetian

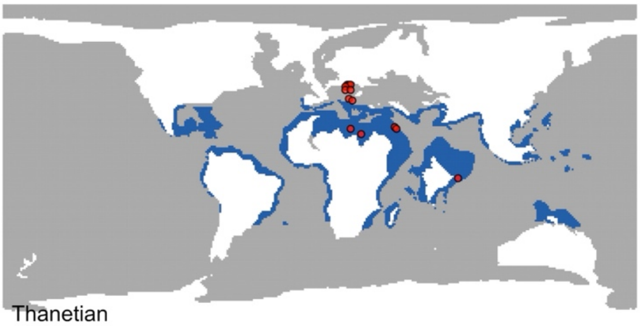

Thanetian

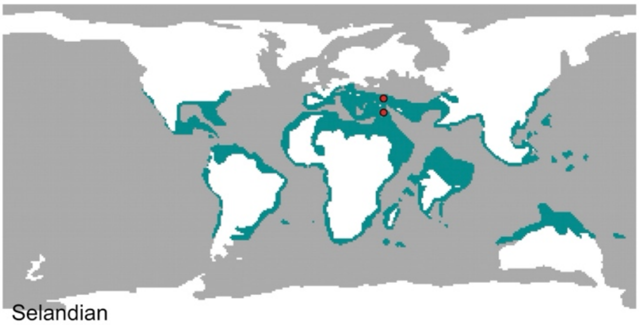

Selandian

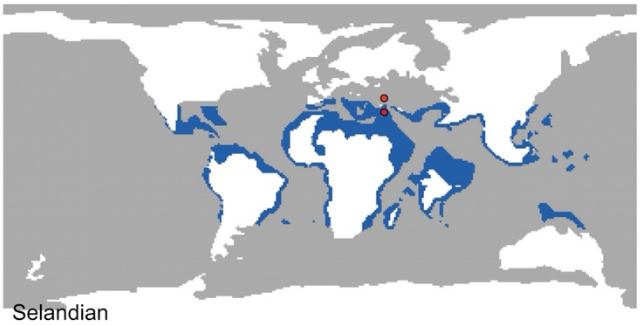

Selandian

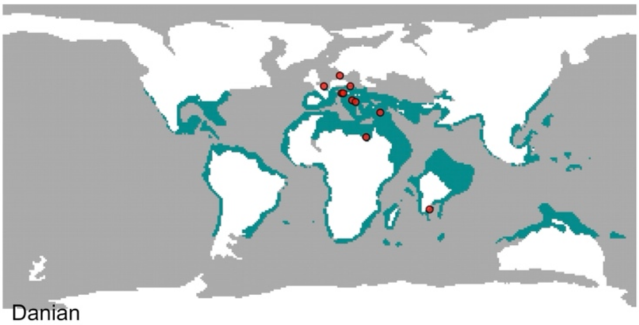

Danian

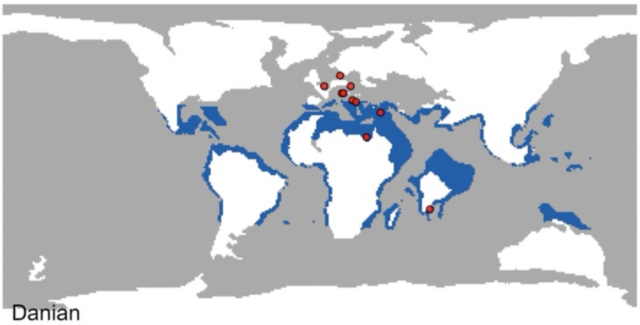

Danian

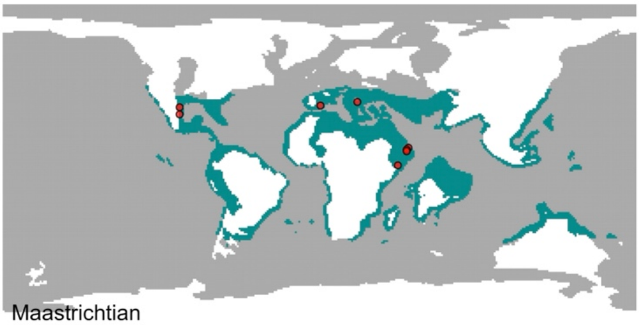

Maastrichtian

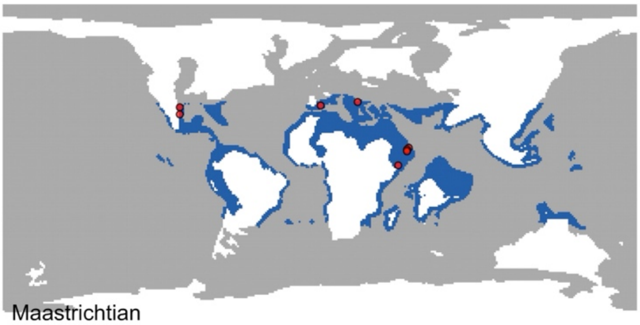

Maastrichtian

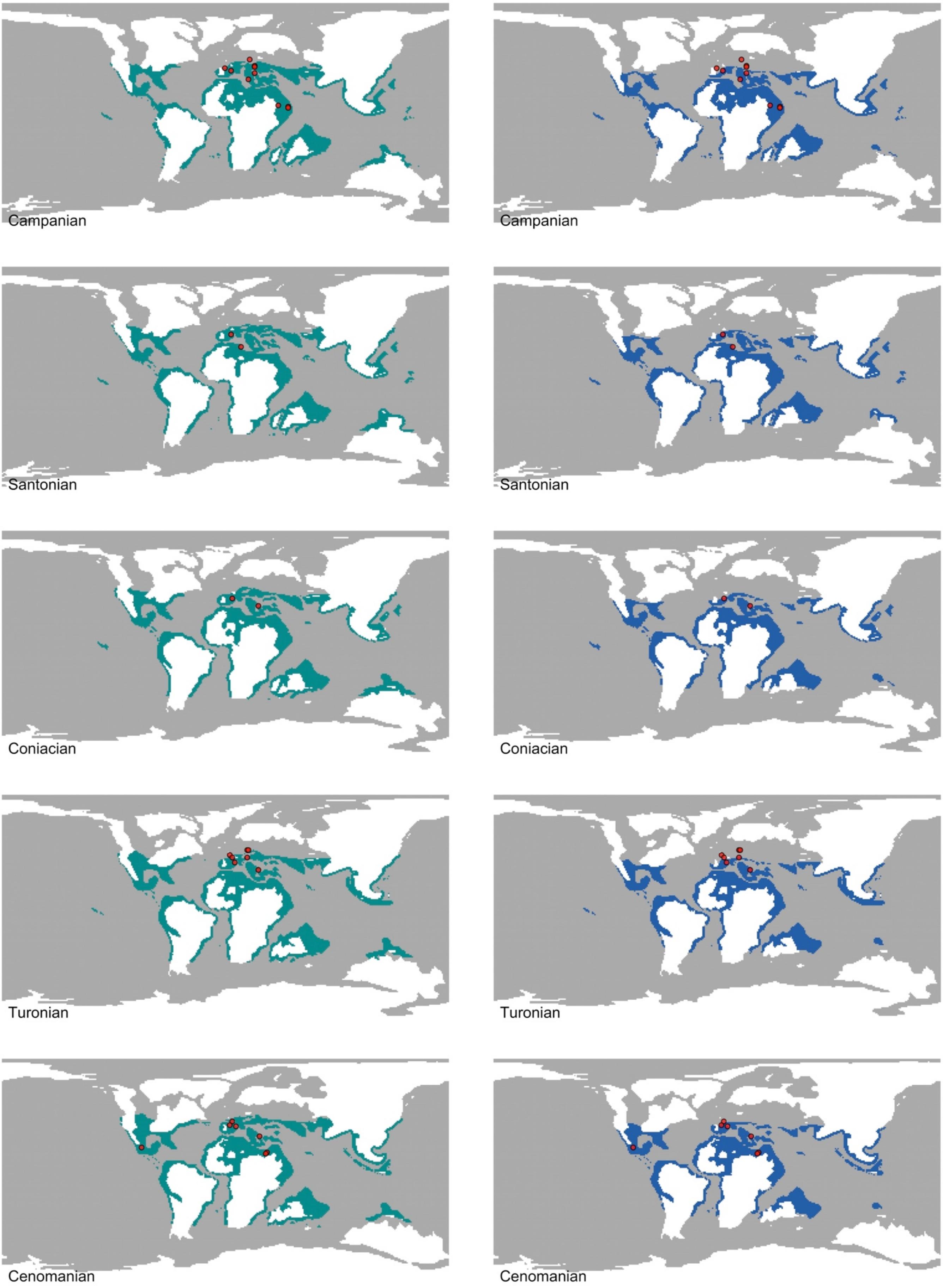
Campanian
Campanian
Santonian
Santonian
Coniacian
Coniacian
Turonian
Turonian
Cenomanian
Cenomanian

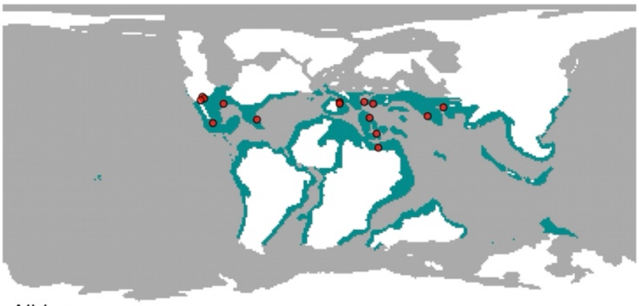

Albian

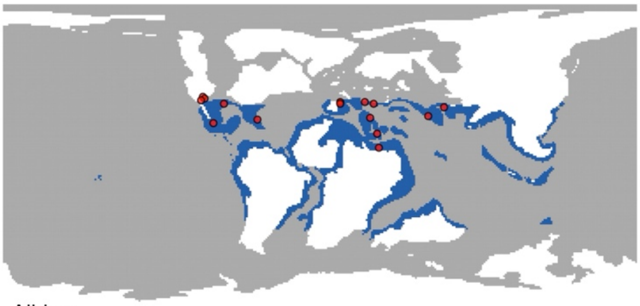

Albian

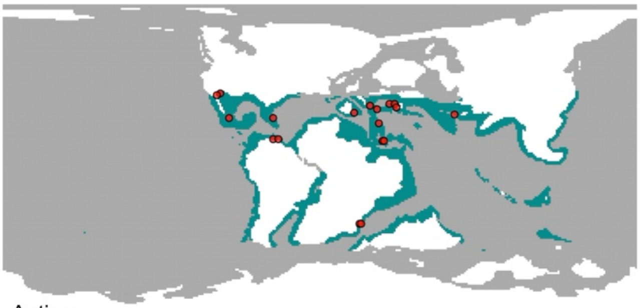

Aptian

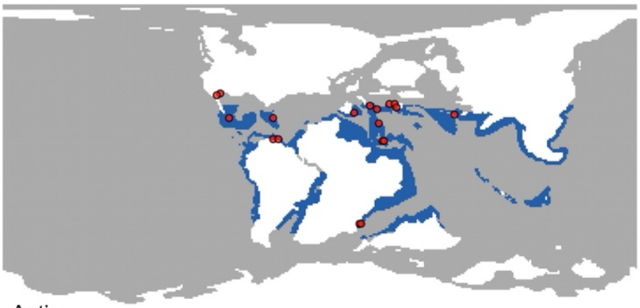

Aptian

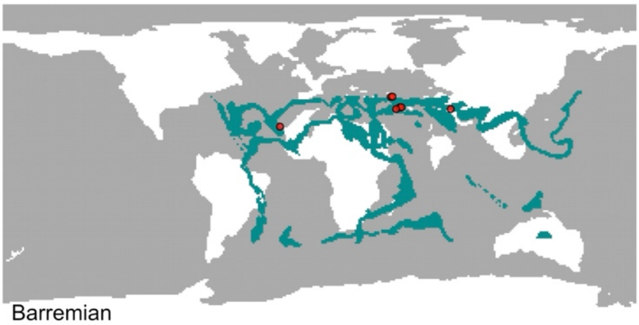

Barremian

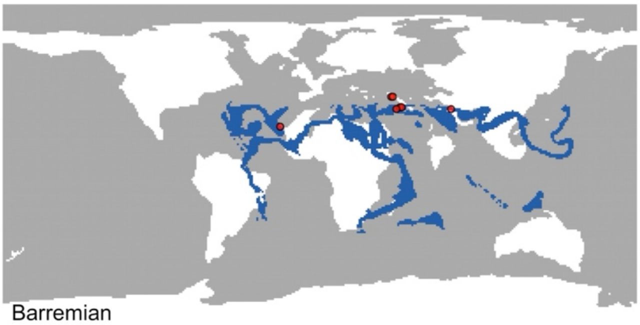

Barremian

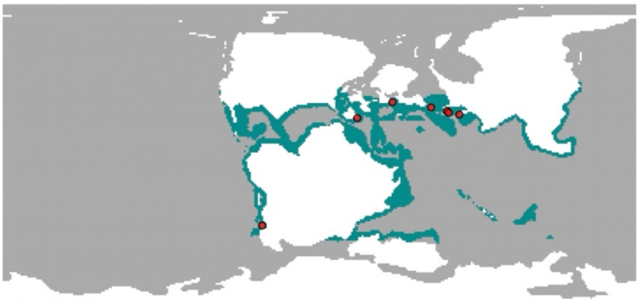

Hauterivian

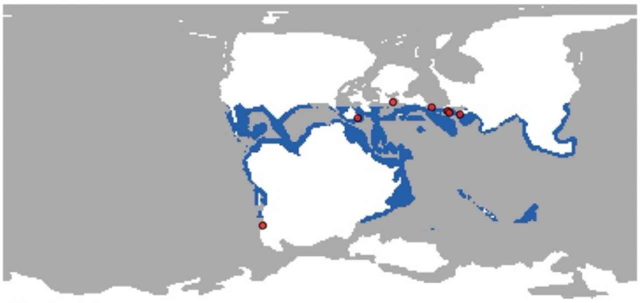

Hauterivian

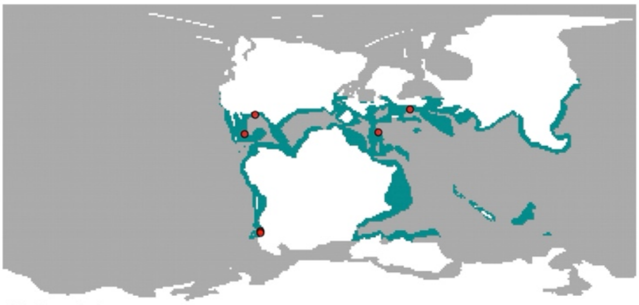

Valanginian

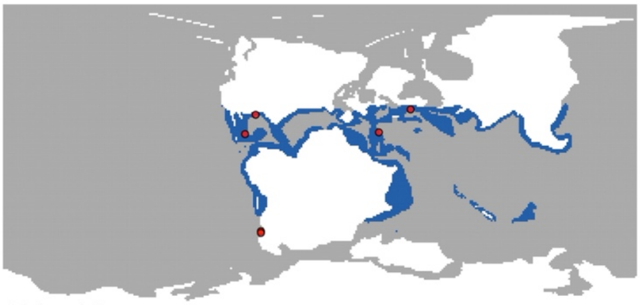

Valanginian

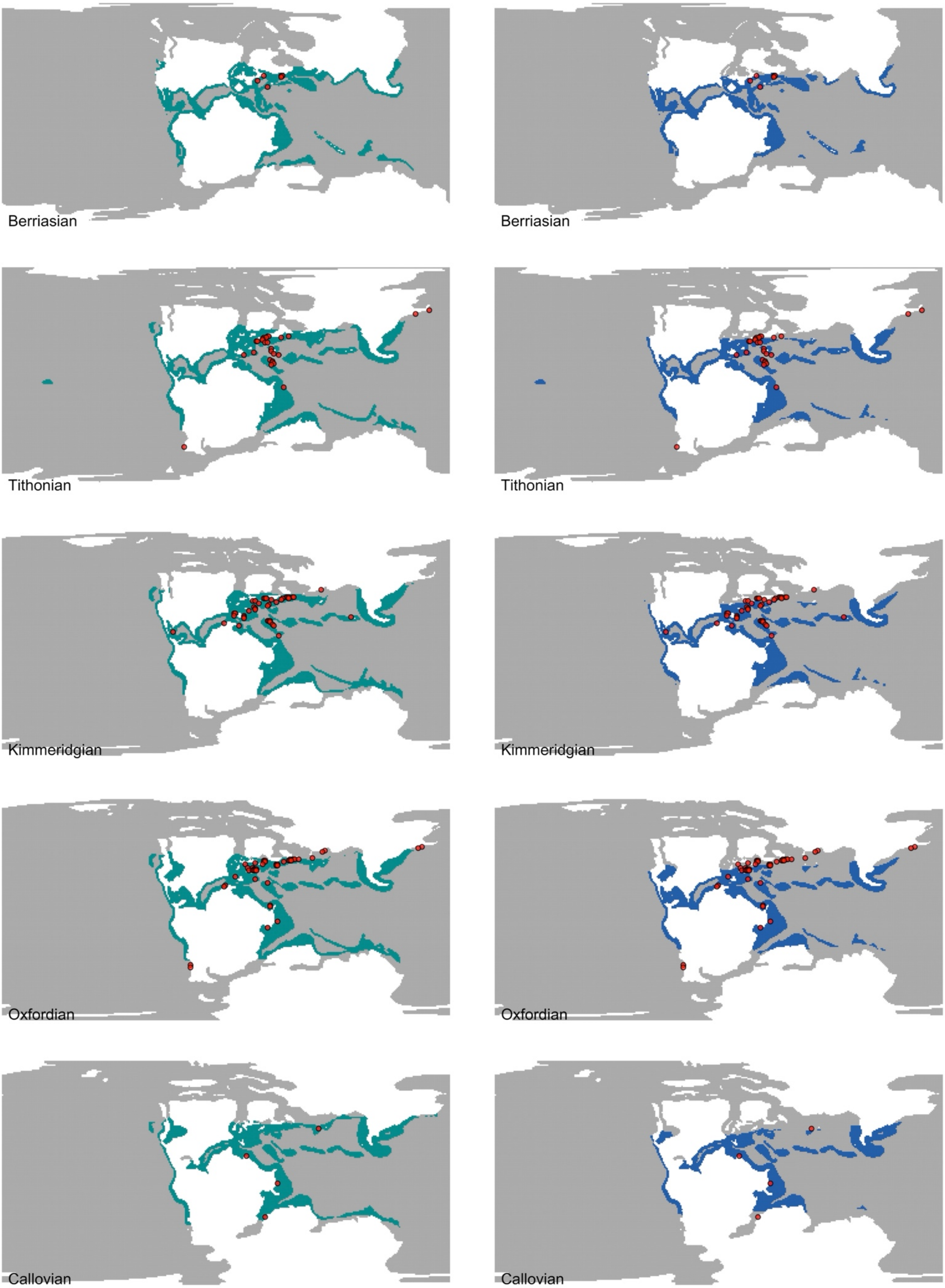
Berriasian
Berriasian
Tithonian
Tithonian
Kimmeridgian
Kimmeridgian
Oxfordian
Oxfordian
Callovian
Callovian

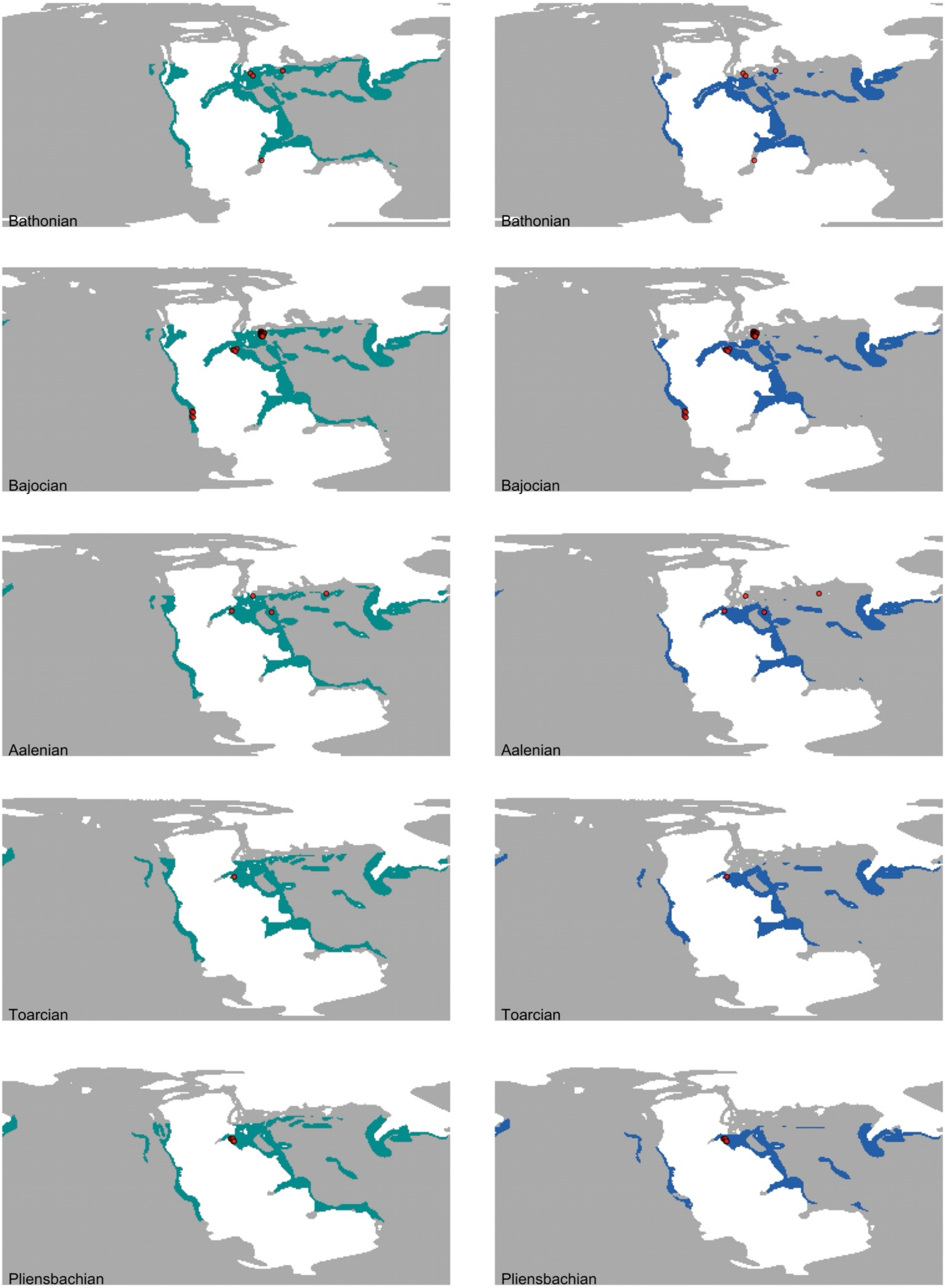
Bathonian
Bathonian
Bajocian
Bajocian
Aalenian
Aalenian
Toarcian
Toarcian
Pliensbachian
Pliensbachian

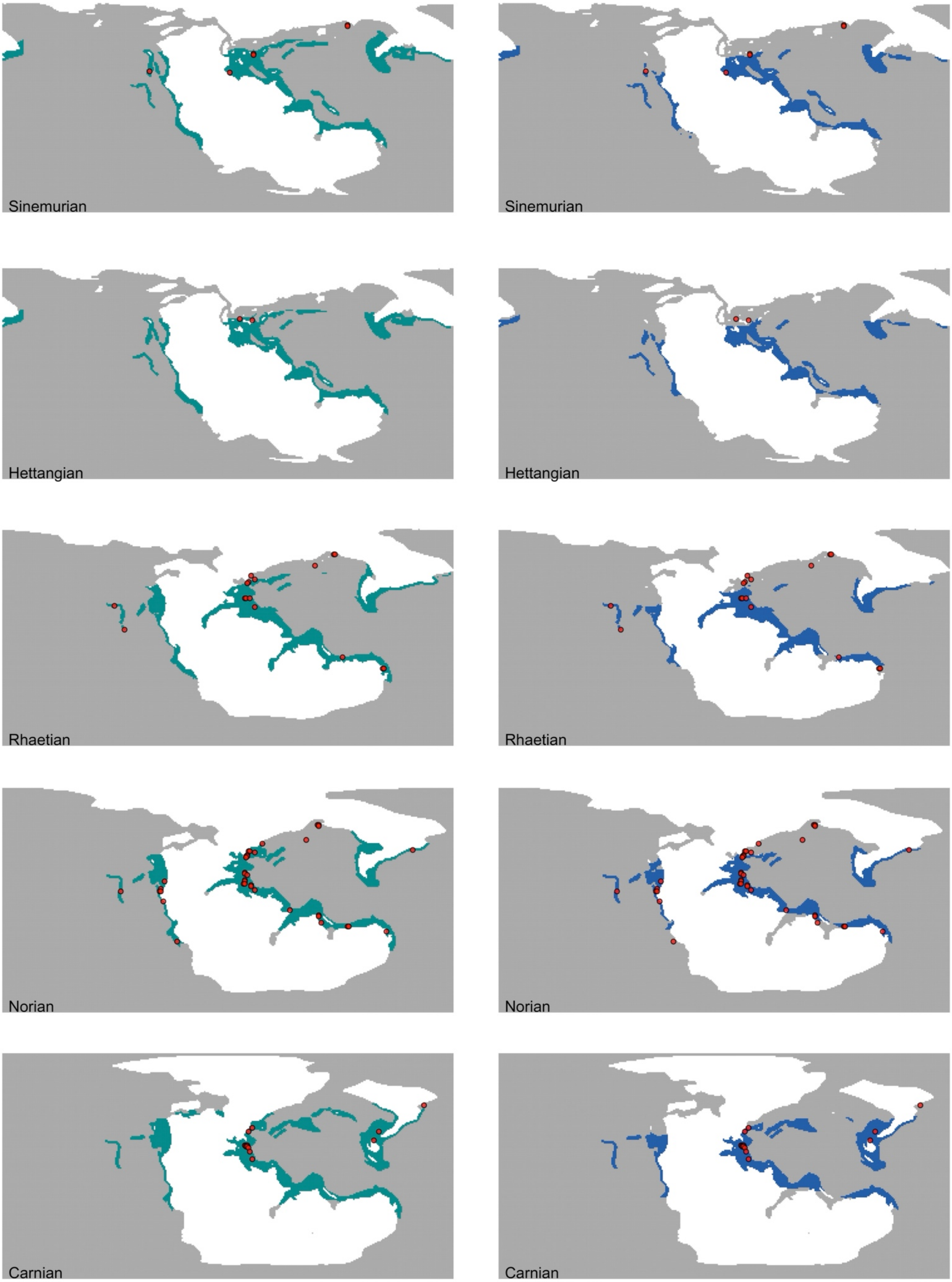
Sinemurian
Sinemurian
Hettangian
Hettangian
Rhaetian
Rhaetian
Norian
Norian
Carnian
Carnian

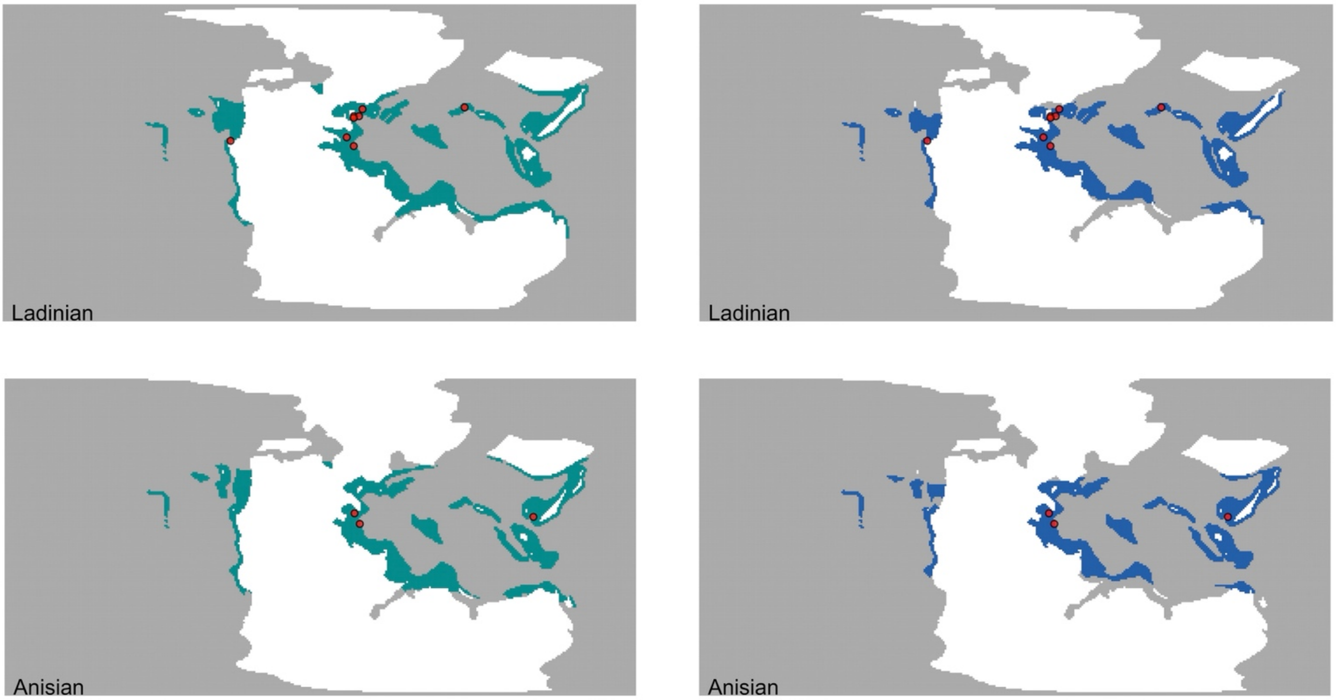

Figure S2. Stage-level (Anisian to Piacenzian) binary habitat suitability maps (suitable/unsuitable) for warm-water coral reefs. Binary maps are converted from continuous suitability predictions using the ‘LTP’ (least training presence) and ‘MaxSSS’ (maximising the sum of sensitivity and specificity) thresholds. LTP predictions are in the left-hand column (suitable locations marked in green), while MaxSSS predictions are in the right-hand column (suitable locations marked in blue). Stage-level hindcasts are based on a modern-calibrated MaxEnt model. Red points indicate the location of known fossil coral reef localities in their respective stage-level palaeorotation.

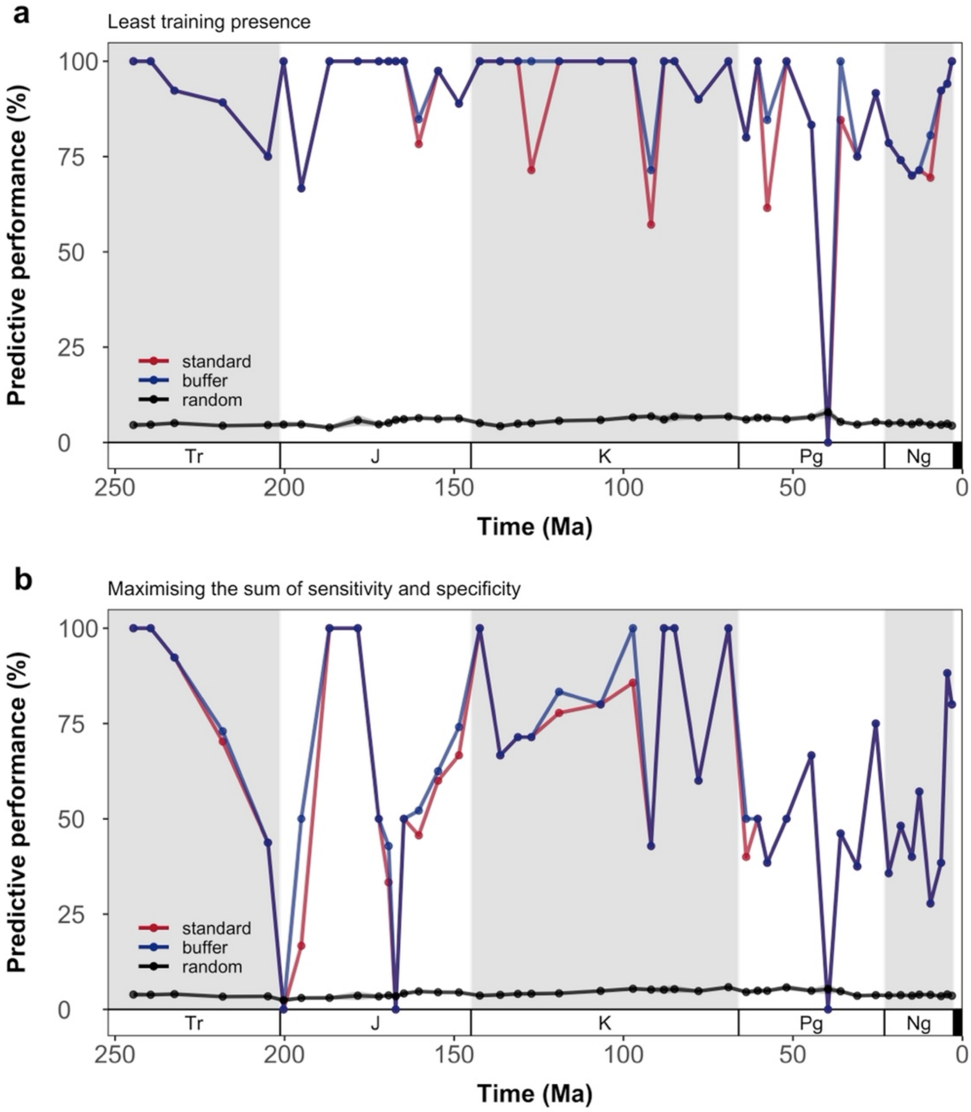

Figure S3. Predictive performance of stage-level (Anisian to Piacenzian) binary hindcasts from habitat suitability modelling for binary thresholds: (a) 'LTP' (least training presence) and (b) 'MaxSSS' (maximising the sum on sensitivity and specificity). Predictive success of fossil reef localities (percentage of total fossil coral reef localities intersecting with cells predicted to be suitable) is indicated by the red points; predictive success of fossil reef localities with a one-cell buffer (one-cell queen moves) is indicated by blue points; predictive success from random point generation is indicated by the black points, along with the 95% confidence intervals. Period abbreviations are as follows: Triassic (Tr), Jurassic (J), Cretaceous (K), Paleogene (Pg), and Neogene (Ng).

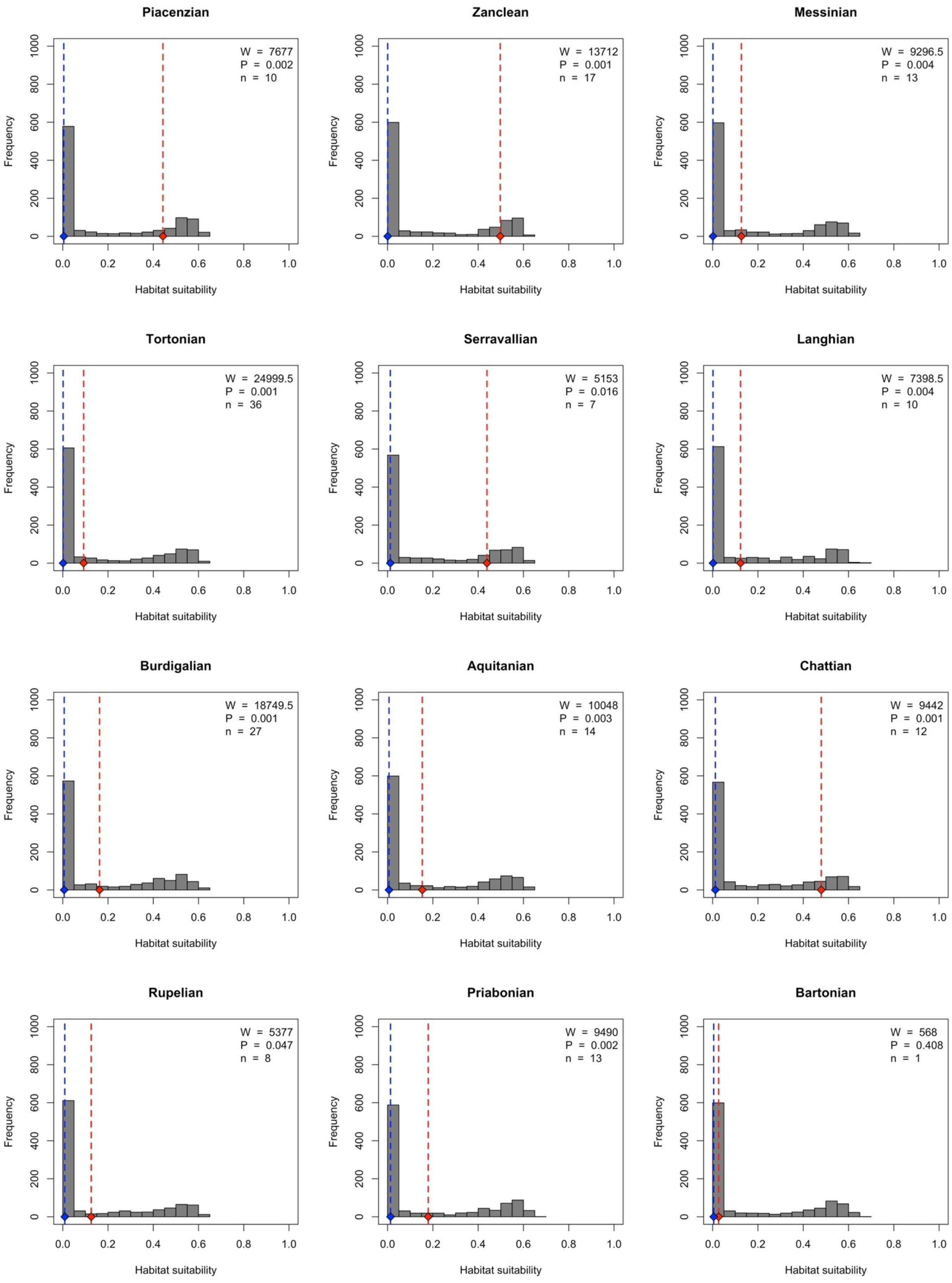
Piacenzian
W = 7677
P = 0.002
n = 10
Zanclean
W = 13712
P = 0.001
n = 17
Messinian
W = 9296.5
P = 0.004
n = 13
Tortonian
W = 24999.5
P = 0.001
n = 36
Serravallian
W = 5153
P = 0.016
n = 7
Langhian
W = 7398.5
P = 0.004
n = 10
Burdigalian
W = 18749.5
P = 0.001
n = 27
Aquitanian
W = 10048
P = 0.003
n = 14
Chattian
W = 9442
P = 0.001
n = 12
Rupelian
W = 5377
P = 0.047
n = 8
Priabonian
W = 9490
P = 0.002
n = 13
Bartonian
W = 568
P = 0.408
n = 1
Frequency
0
200
400
600
800
1000
Habitat suitability
0.0
0.2
0.4
0.6
0.8
1.0

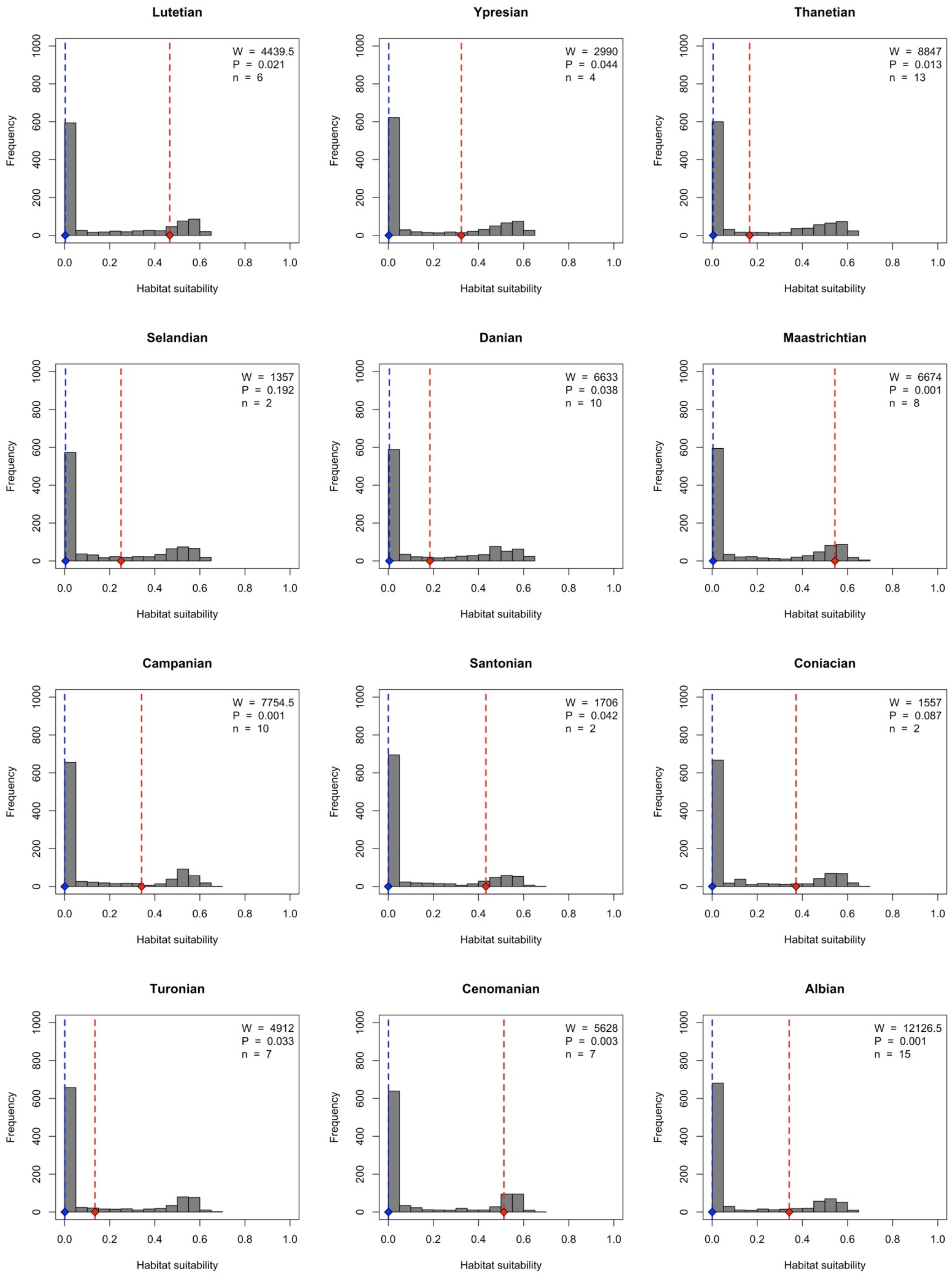
Lutetian
W = 4439.5
P = 0.021
n = 6
Ypresian
W = 2990
P = 0.044
n = 4
Thanetian
W = 8847
P = 0.013
n = 13
Selandian
W = 1357
P = 0.192
n = 2
Danian
W = 6633
P = 0.038
n = 10
Maastrichtian
W = 6674
P = 0.001
n = 8
Campanian
W = 7754.5
P = 0.001
n = 10
Santonian
W = 1706
P = 0.042
n = 2
Coniacian
W = 1557
P = 0.087
n = 2
Turonian
W = 4912
P = 0.033
n = 7
Cenomanian
W = 5628
P = 0.003
n = 7
Albian
W = 12126.5
P = 0.001
n = 15
Frequency
Habitat suitability
0
200
400
600
800
1000
0.0
0.2
0.4
0.6
0.8
1.0

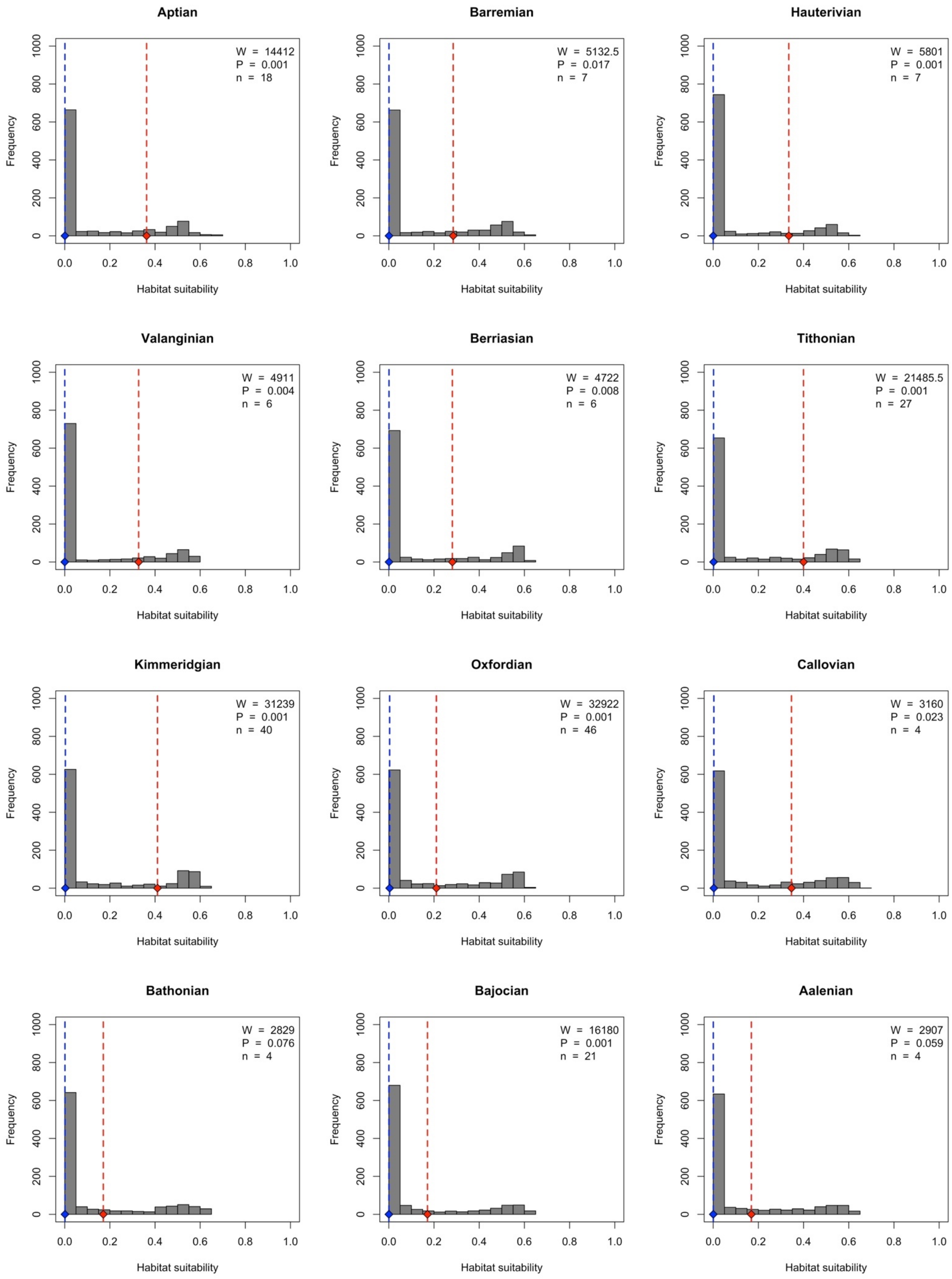
Aptian
W = 14412
P = 0.001
n = 18
Barremian
W = 5132.5
P = 0.017
n = 7
Hauterivian
W = 5801
P = 0.001
n = 7
Valanginian
W = 4911
P = 0.004
n = 6
Berriasian
W = 4722
P = 0.008
n = 6
Tithonian
W = 21485.5
P = 0.001
n = 27
Kimmeridgian
W = 31239
P = 0.001
n = 40
Oxfordian
W = 32922
P = 0.001
n = 46
Callovian
W = 3160
P = 0.023
n = 4
Bathonian
W = 2829
P = 0.076
n = 4
Bajocian
W = 16180
P = 0.001
n = 21
Aalenian
W = 2907
P = 0.059
n = 4
Frequency
Habitat suitability
0 200 400 600 800 1000
0.0 0.2 0.4 0.6 0.8 1.0

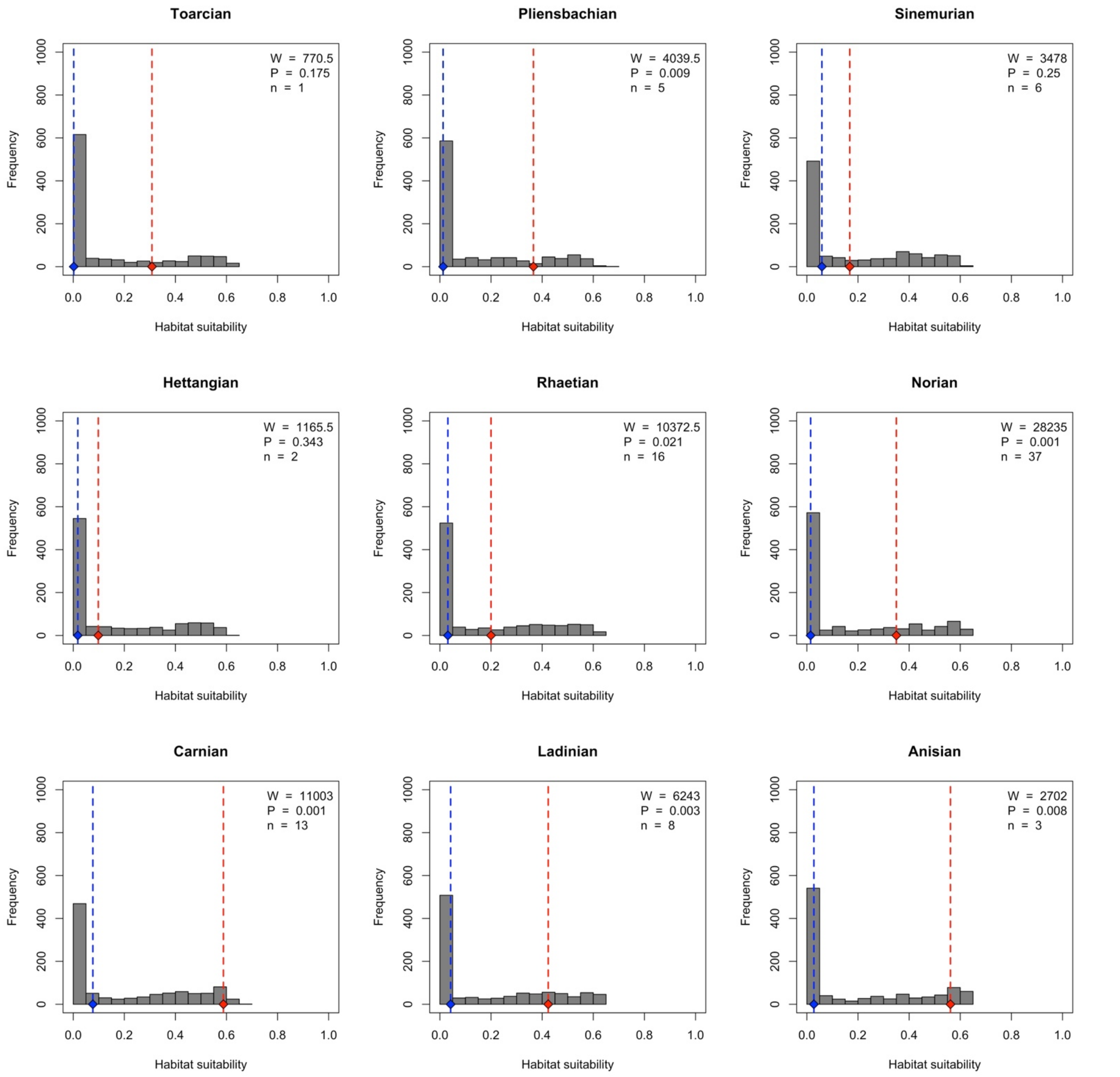

Figure S4. Predictive performance of stage-level (Anisian to Piacenzian) continuous predictions from habitat suitability modelling. Results show the distribution of continuous suitability values within hindcast predictions (grey bars), the median suitability value associated with known fossil coral reef localities (red diamond), and the median suitability value associated with randomly generated points (*n* = 1,000; blue diamond). The number of fossil coral reef localities for each stage is depicted in the top-right corner of each plot (n). Two-sample Wilcoxon signed-rank test results (W statistic; P-value; top-right corner) indicate that for the majority of stages (37/45), suitability values associated with fossil coral reef localities are significantly greater than those of randomly generated points.

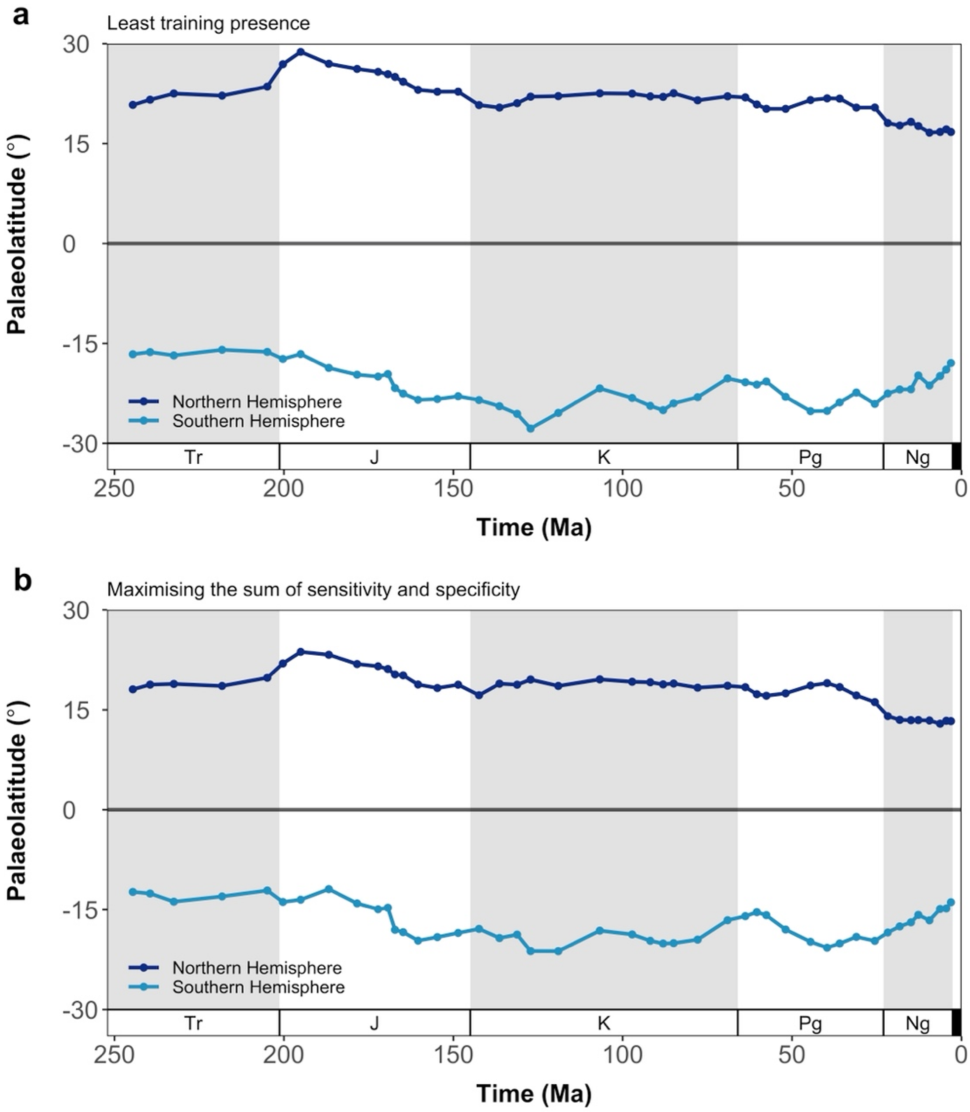

Figure S5. Palaeolatitudinal shifts in the centroid of suitable habitats for warm-water coral reefs from the Anisian (Triassic) to the Piacenizan (Neogene). The centroid was computed from binary suitability maps under two thresholds: (a) 'LTP' (least training presence) and (b) 'MaxSSS' (maximising the sum of sensitivity and specificity). Centroid calculation was carried out for each hemisphere and implemented with weights proportional to the area of each suitable cell to account for variable cell area with latitude. Period abbreviations are as follows: Triassic (Tr), Jurassic (J), Cretaceous (K), Paleogene (Pg), and Neogene (Ng).

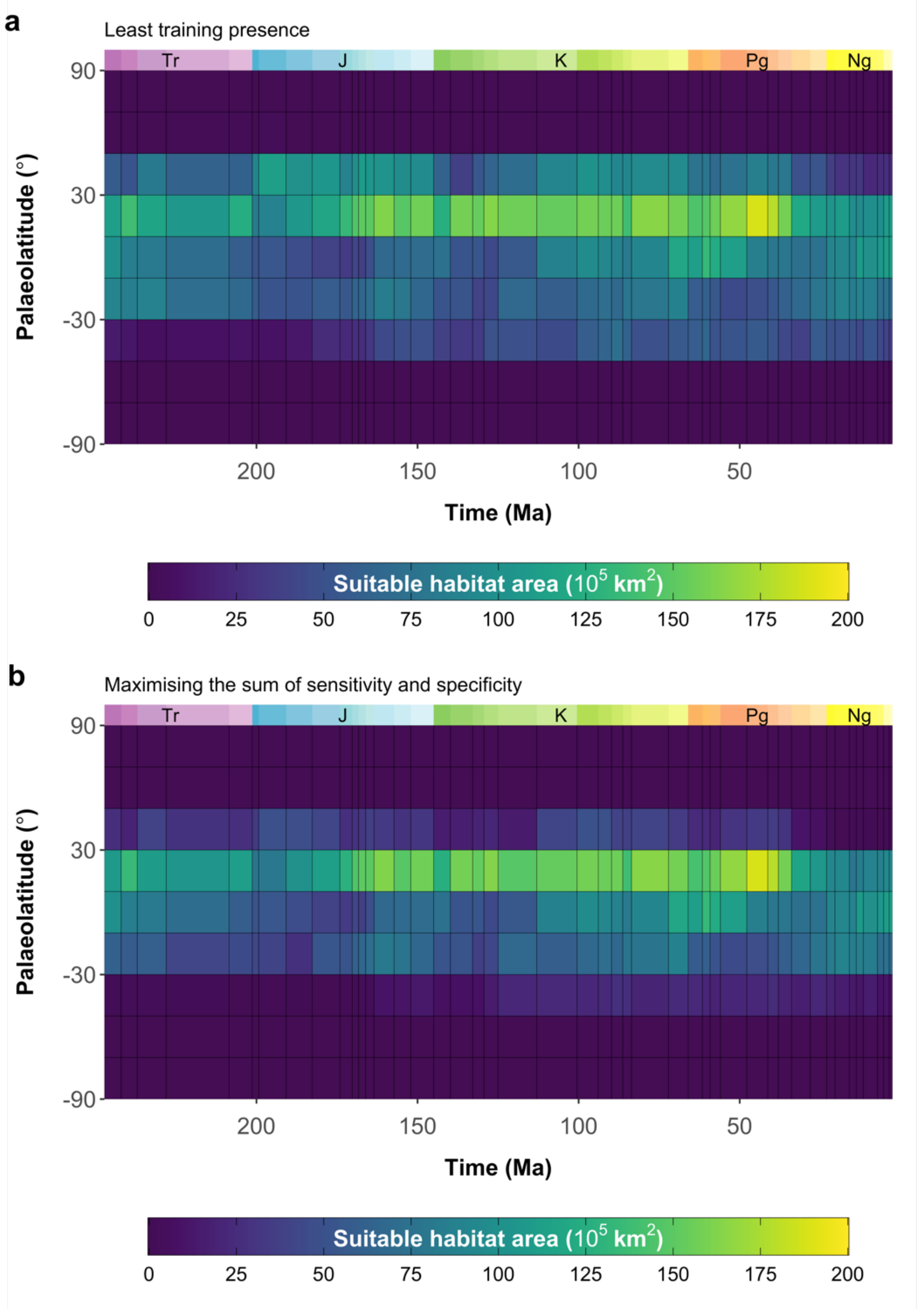

Figure S6. Palaeolatitudinal (20° bins) estimates of suitable habitat area (Anisian to Piacenzian) for warm-water coral reefs under two binary thresholds: (a) 'LTP' (least training presence) and (b) 'MaxSSS' (maximising the sum of sensitivity and specificity). Stage-level hindcasts are based on a modern-calibrated MaxEnt model, and area is calculated from binary predictions (LTP/MaxSSS). Period abbreviations are as follows: Triassic (Tr), Jurassic (J), Cretaceous (K), Paleogene (Pg), and Neogene (Ng).

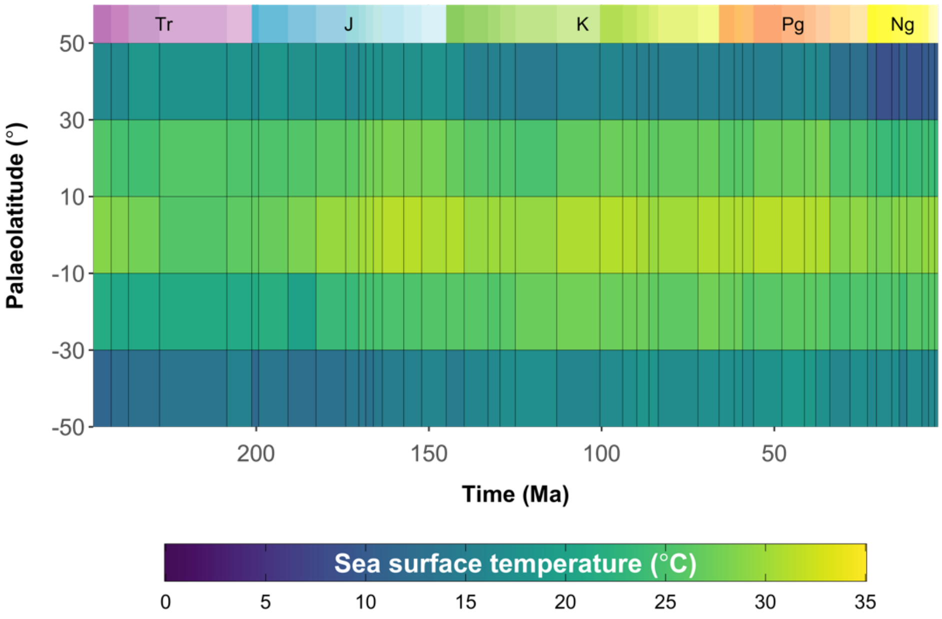

Figure S7. Stage-level (Anisian–Piacenzian) palaeolatitudinal (20° bins) mean minimum sea surface temperatures from the HadCM3L (HadCM3BL-M2.1aE) climate model. Note the decline in sea surface temperature from the Priabonian (Paleogene) towards the present-day. Period abbreviations are as follows: Triassic (Tr), Jurassic (J), Cretaceous (K), Paleogene (Pg), and Neogene (Ng).

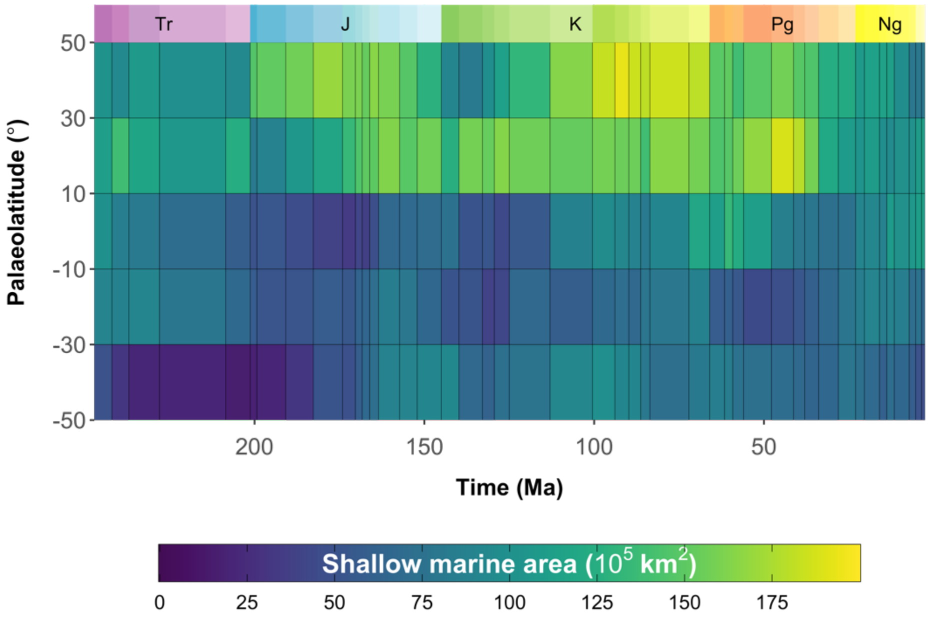

Figure S8. Stage-level (Anisian–Piacenzian) palaeolatitudinal (20° bins) shallow marine substrate area from Getech's digital elevation models. Shallow marine area is defined as cells with substrate depth less than 200 m, which approximates the photic zone. Note the decline in shallow marine substrate across the Jurassic/Cretaceous boundary at 30–50°N, as well as the increase in the tropics/subtropics from the Priabonian (Paleogene) towards the present day. Period abbreviations are as follows: Triassic (Tr), Jurassic (J), Cretaceous (K), Paleogene (Pg), and Neogene (Ng).

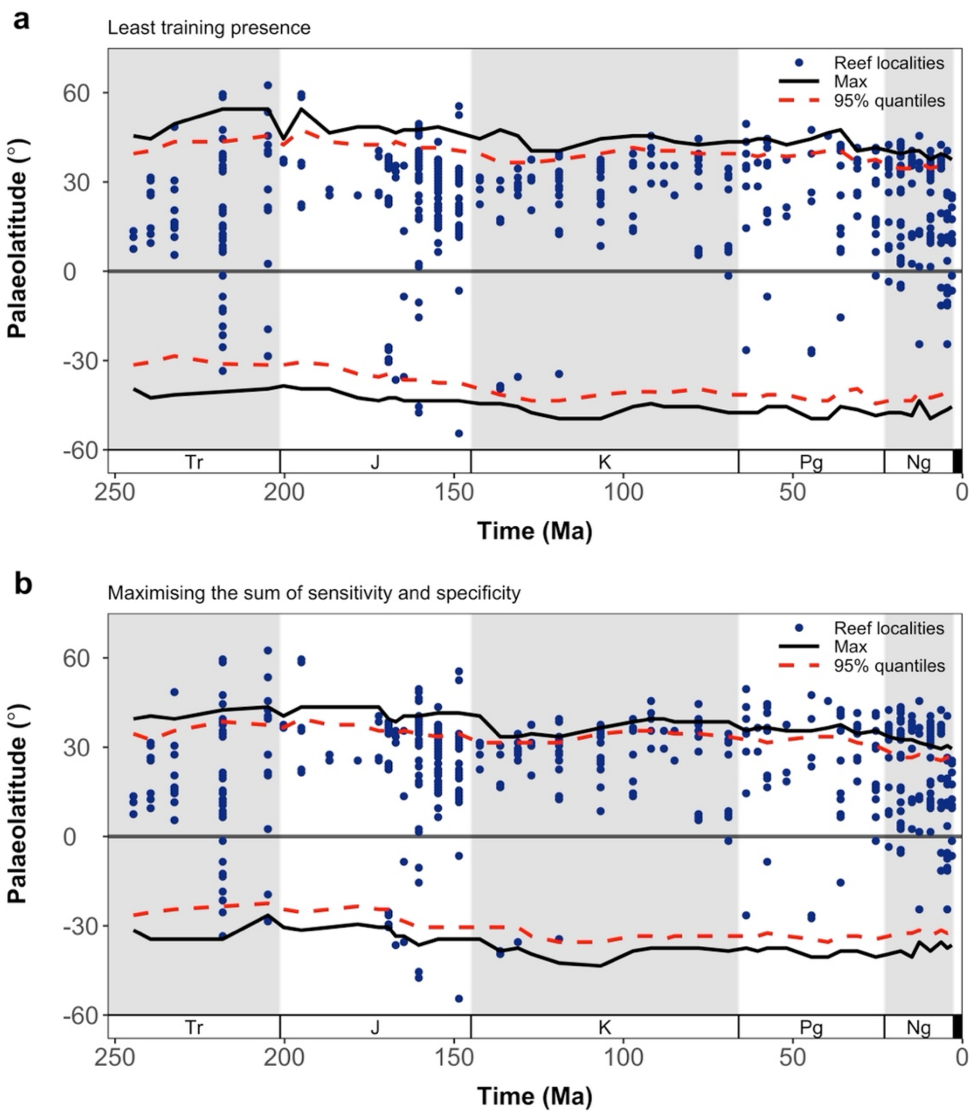

Figure S9. Stage-level estimates of warm-water coral reef zone from the Anisian (Triassic) to the Piacenzian (Neogene). Reef zone (black lines) is defined as the most poleward palaeolatitude of suitable habitat in each hemisphere (North and South) under two binary thresholds: (a) 'LTP' (least training presence) and (b) 'MaxSSS' (maximising the sum of sensitivity and specificity). The 95% quantiles of estimated stage-level reef zone are depicted by the dashed red line, while fossil coral reef localities are indicated by the blue points. Period abbreviations are as follows: Triassic (Tr), Jurassic (J), Cretaceous (K), Paleogene (Pg), and Neogene (Ng).

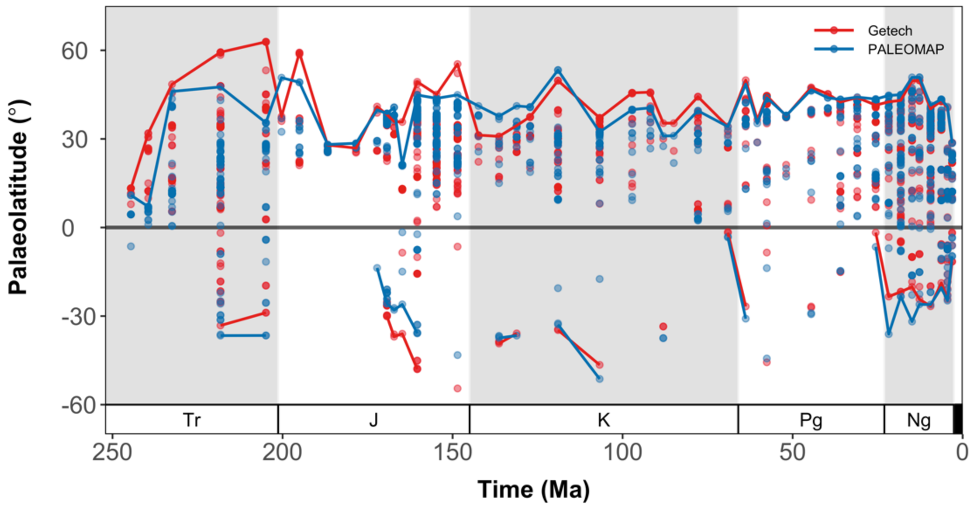

Figure S10. Palaeolatitudinal plot of fossil coral reefs in their stage-level rotation. Reconstructed palaeolatitudes using the Getech (red) and PALEOMAP (blue) palaeo-plate rotation models are shown. Fossil reefs are palaeorotated to the midpoint age of their respective stage-level bins. Points represent individual fossil coral reefs used in this study. Solid lines indicate the extent of the most poleward fossil coral reef in the Northern and Southern Hemisphere. Period abbreviations are as follows: Triassic (Tr), Jurassic (J), Cretaceous (K), Paleogene (Pg), and Neogene (Ng).

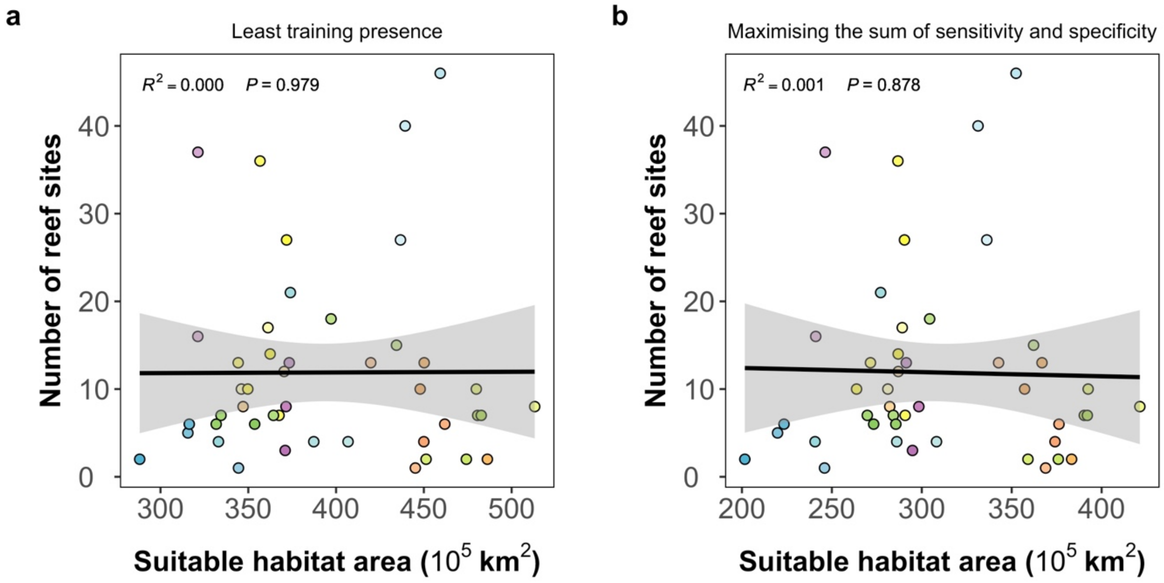

Figure S11. Relationship between the number of reef sites ('true coral reefs') and the estimated suitable habitat area for each geological stage, from the Anisian (Triassic) to the Piacenzian (Neogene). (a) Relationship between the number of reef sites ('true coral reefs') and the estimated suitable habitat area under binary threshold 'least training presence' (LTP). (b) Relationship between the number of reef sites ('true coral reefs') and the estimated suitable habitat area under binary threshold 'maximising the sum of sensitivity and specificity' (MaxSSS). Ordinary least-squares regression analyses suggests that there is no significant relationship between the number of fossil coral reef sites and the availability of climatically suitable habitat. The coefficient of determination (R^2) with associated P-values are depicted within each plot. Each point represents an individual stage and is coloured accordingly.

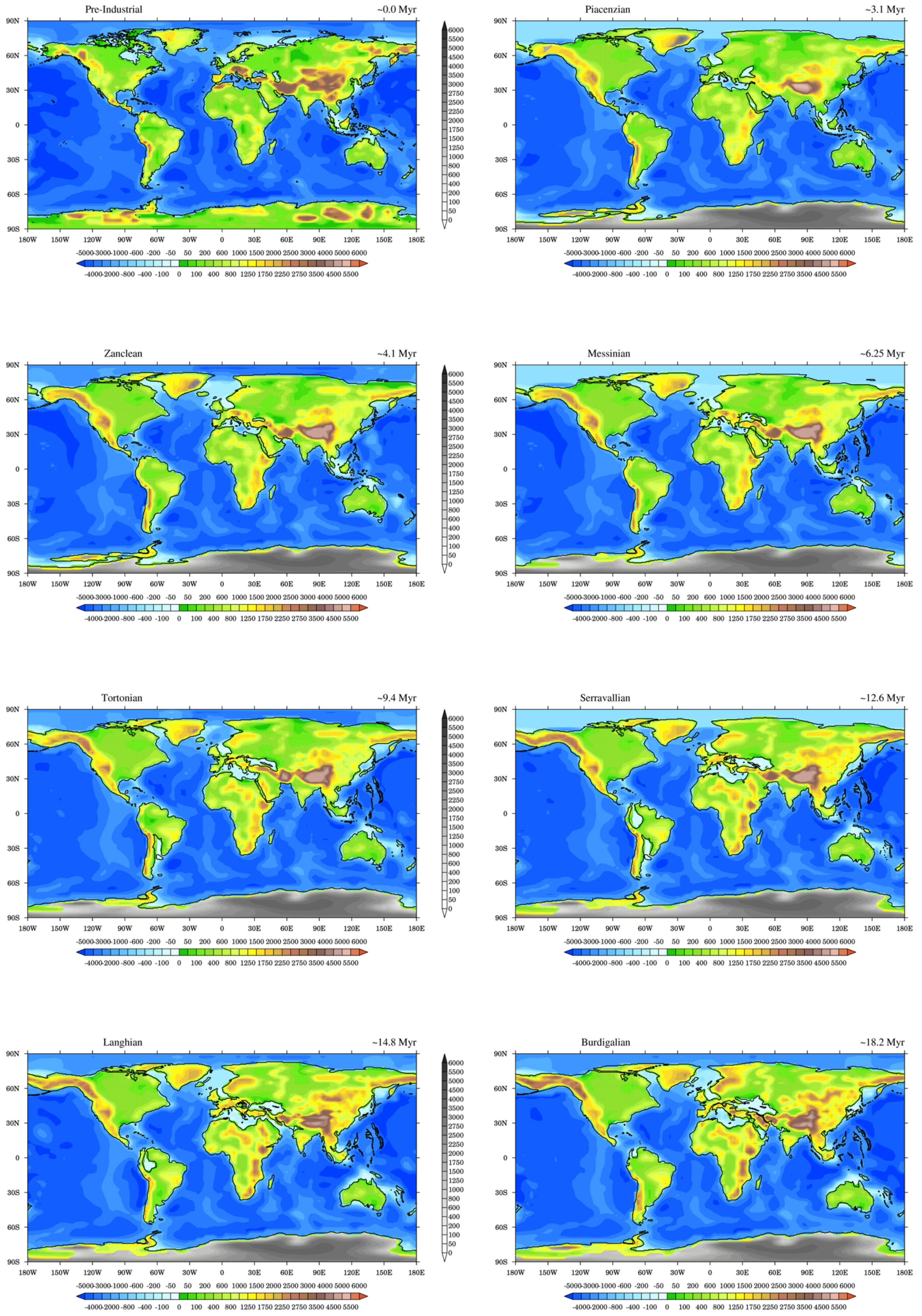
Pre-Industrial
~0.0 Myr
Piacenzian
~3.1 Myr
Zanclean
~4.1 Myr
Messinian
~6.25 Myr
Tortonian
~9.4 Myr
Serravallian
~12.6 Myr
Langhian
~14.8 Myr
Burdigalian
~18.2 Myr
90N 60N 30N 0 30S 60S 90S
180W 150W 120W 90W 60W 30W 0 30E 60E 90E 120E 150E 180E
-5000 -3000 -1000 -600 -200 -50 50 200 600 1000 1500 2000 2500 3000 4000 5000 6000
-4000 -2000 -800 -400 -100 0 100 400 800 1250 1750 2250 2750 3500 4500 5500
6000 5500 5000 4500 4000 3500 3000 2750 2500 2250 2000 1750 1500 1250 1000 800 600 400 200 100 50 0

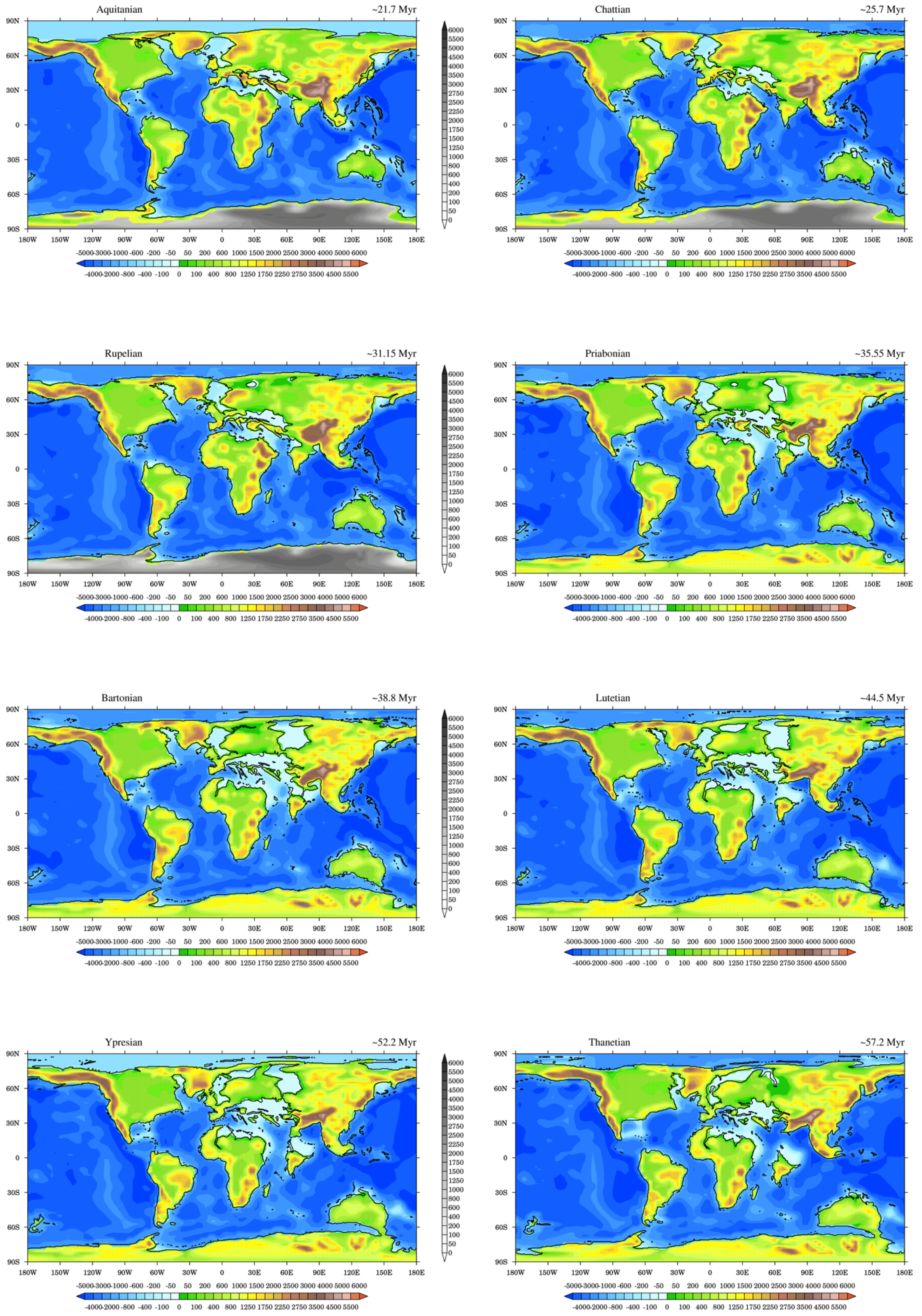
Aquitanian
~21.7 Myr
Chattian
~25.7 Myr
Rupelian
~31.15 Myr
Priabonian
~35.55 Myr
Bartonian
~38.8 Myr
Lutetian
~44.5 Myr
Ypresian
~52.2 Myr
Thanetian
~57.2 Myr

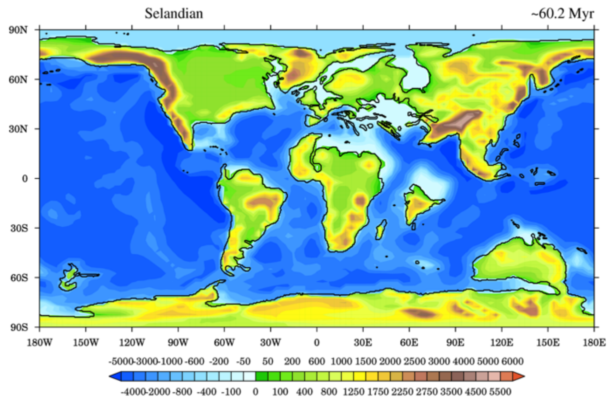
Selandian
~60.2 Myr

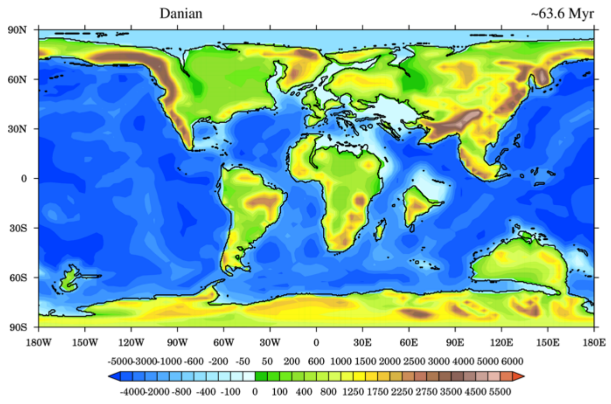
Danian
~63.6 Myr

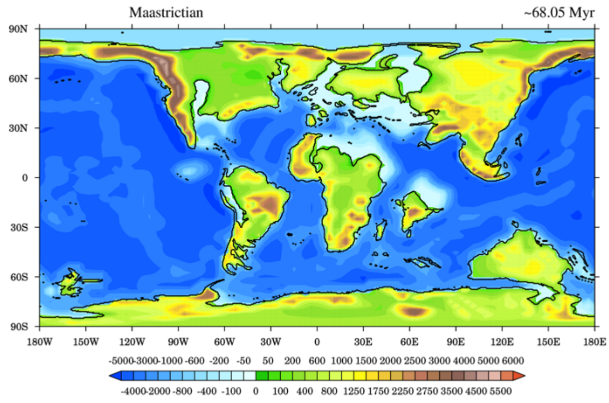
Maastrictian
~68.05 Myr

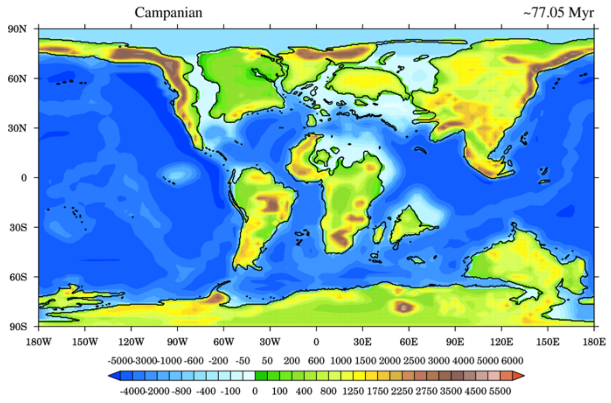
Campanian
~77.05 Myr

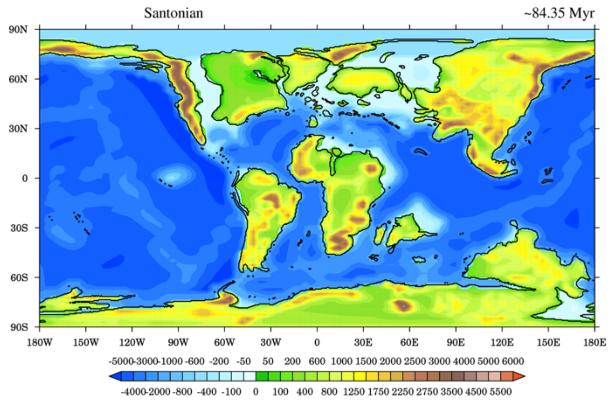
Santonian
~84.35 Myr

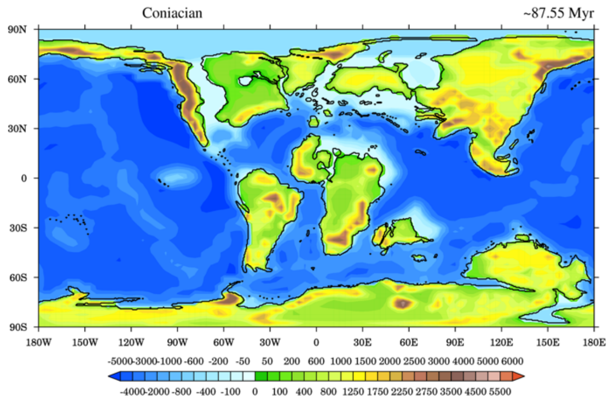
Coniacian
~87.55 Myr

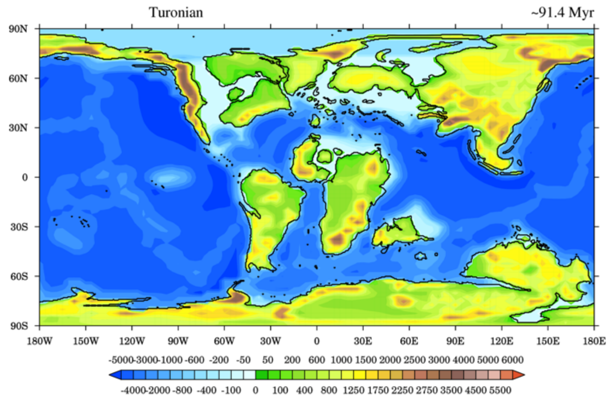
Turonian
~91.4 Myr

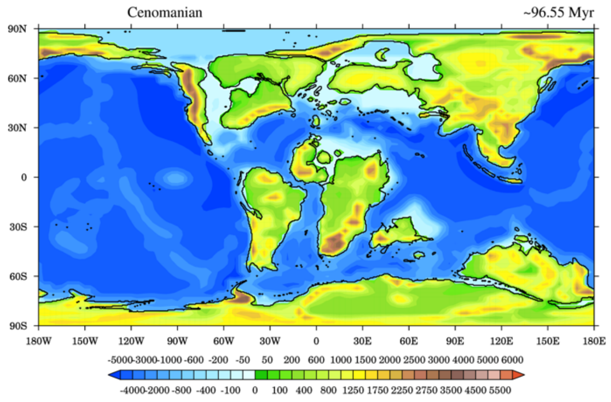
Cenomanian
~96.55 Myr

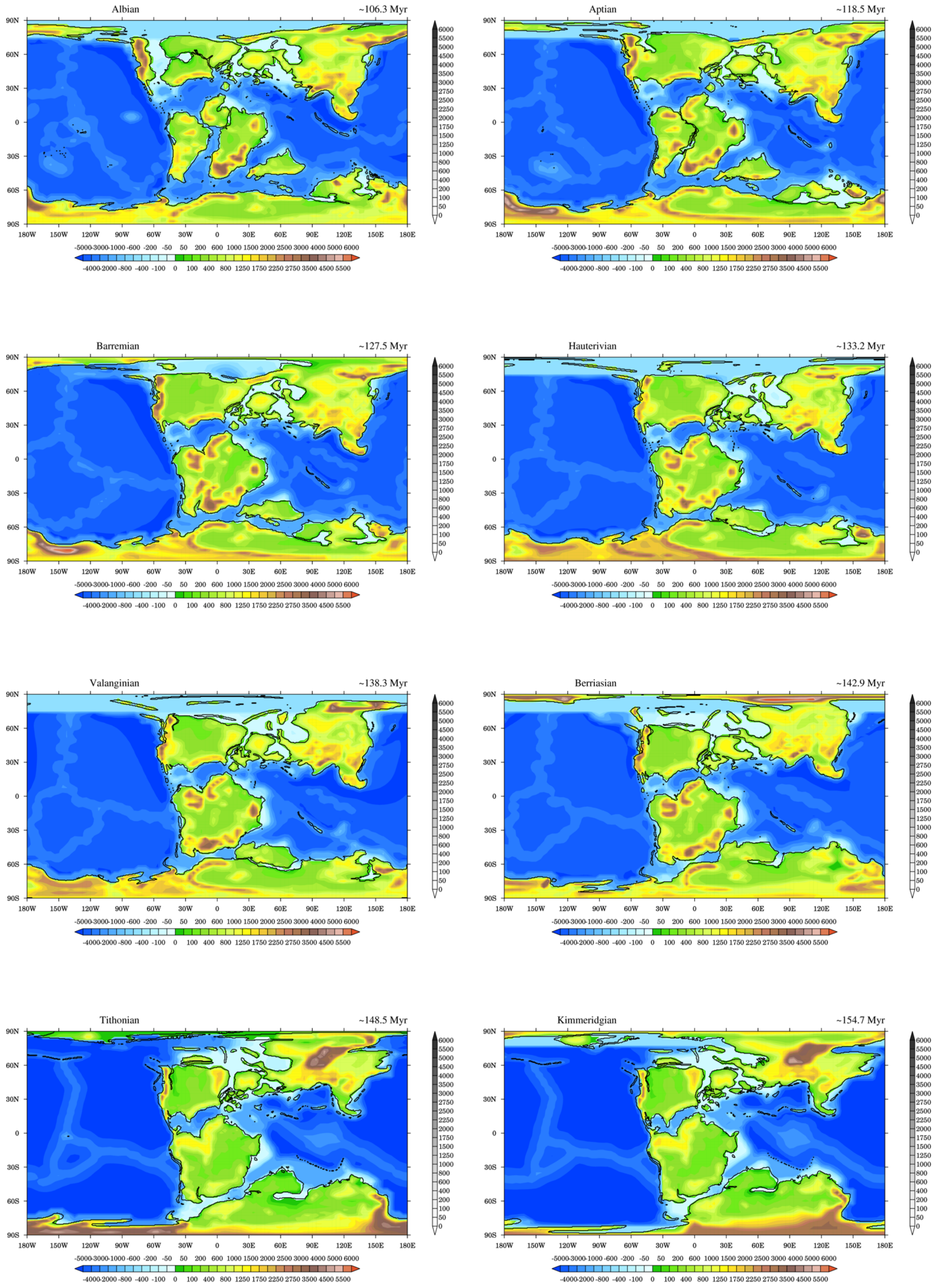
Albian
~106.3 Myr
Aptian
~118.5 Myr
Barremian
~127.5 Myr
Hauterivian
~133.2 Myr
Valanginian
~138.3 Myr
Berriasian
~142.9 Myr
Tithonian
~148.5 Myr
Kimmeridgian
~154.7 Myr

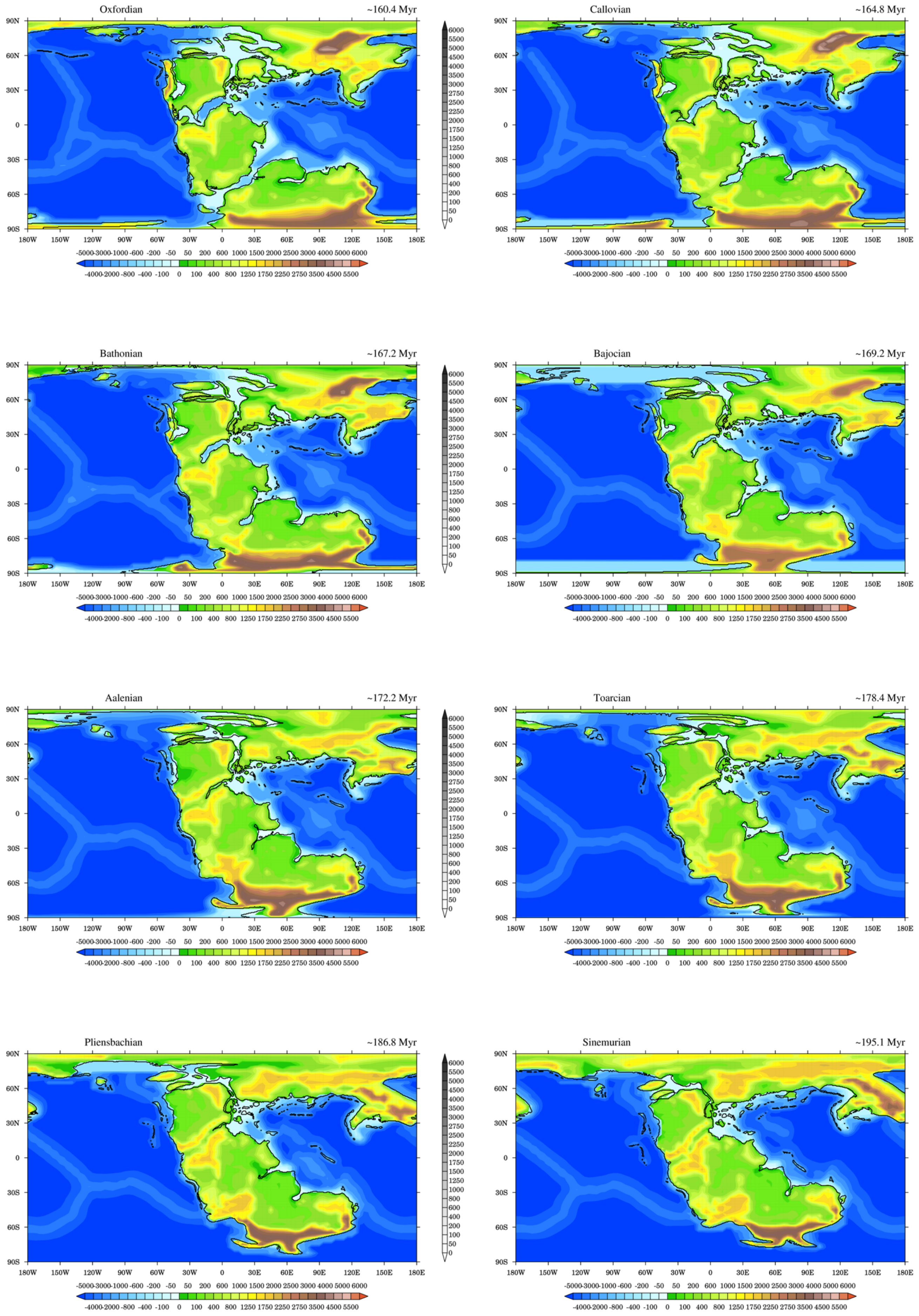
Oxfordian
~160.4 Myr
Callovian
~164.8 Myr
Bathonian
~167.2 Myr
Bajocian
~169.2 Myr
Aalenian
~172.2 Myr
Toarcian
~178.4 Myr
Pliensbachian
~186.8 Myr
Sinemurian
~195.1 Myr
90N 60N 30N 0 30S 60S 90S
180W 150W 120W 90W 60W 30W 0 30E 60E 90E 120E 150E 180E
-5000 -3000 -1000 -600 -200 -50 50 200 600 1000 1500 2000 2500 3000 4000 5000 6000
-4000 -2000 -800 -400 -100 0 100 400 800 1250 1750 2250 2750 3500 4500 5500

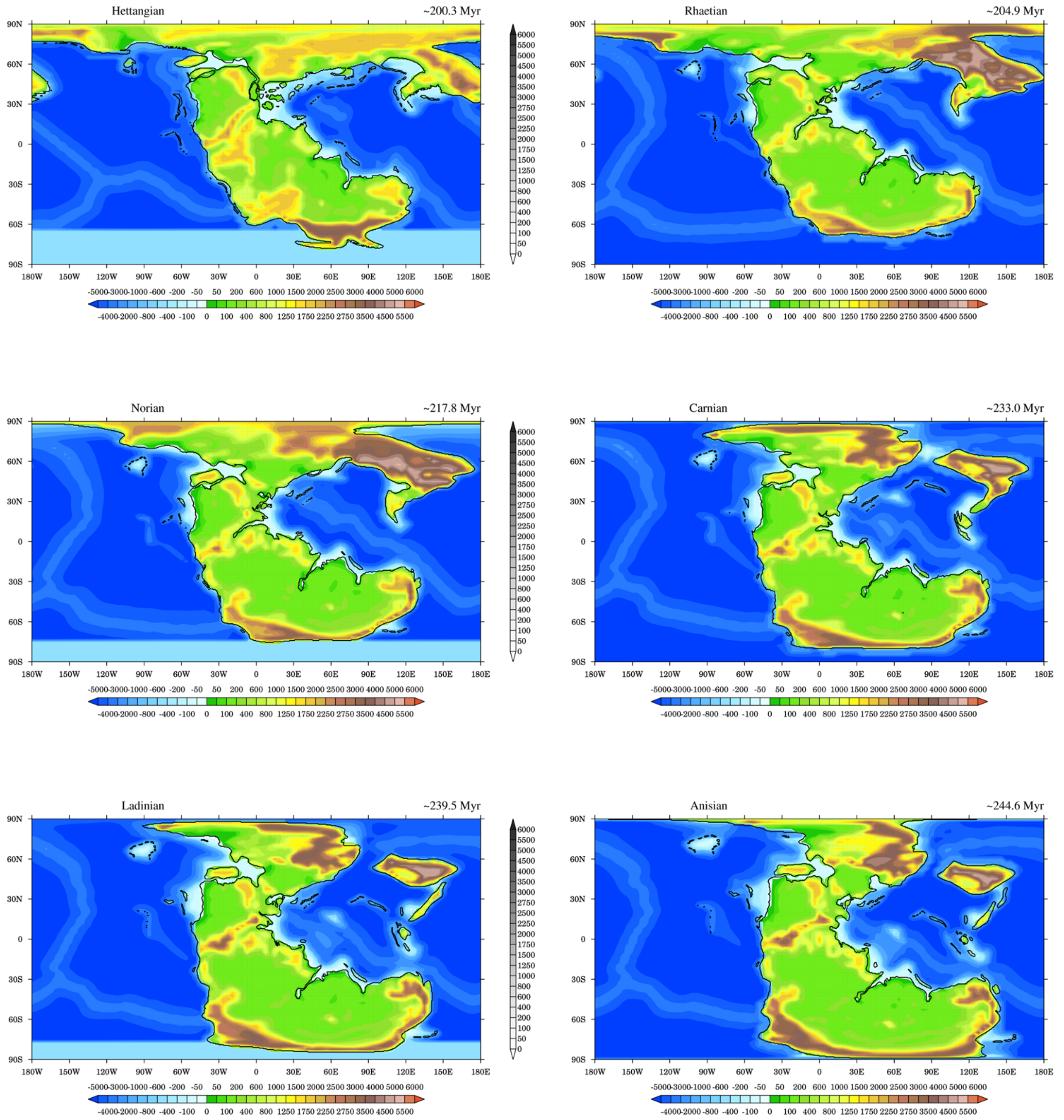

Figure S12. Getech's orography and bathymetry models used in this study. Models are offered at a horizontal resolution of 0.5º x 0.5º and provide the boundary conditions for the HadCM3L climate model simulations. Orography and bathymetry are plotted in metres above/below sea level, with the colour key depicted at the bottom of each plot. Icesheet thickness (in metres) is also depicted in each plot (where applicable) and denoted by the greyscale bar in the right of each plot.
